# Chromatin architecture changes and DNA replication fork collapse are critical features in cryopreserved cells that are differentially controlled by cryoprotectants

**DOI:** 10.1038/s41598-018-32939-5

**Published:** 2018-10-02

**Authors:** Martin Falk, Iva Falková, Olga Kopečná, Alena Bačíková, Eva Pagáčová, Daniel Šimek, Martin Golan, Stanislav Kozubek, Michaela Pekarová, Shelby E. Follett, Bořivoj Klejdus, K. Wade Elliott, Krisztina Varga, Olga Teplá, Irena Kratochvílová

**Affiliations:** 1 The Czech Academy of Sciences, Institute of Biophysics, Královopolská 135, CZ-612 65 Brno, Czech Republic; 2 The Czech Academy of Sciences, Institute of Physics, Na Slovance 2, CZ-182 21 Prague 8, Czech Republic; 3 Faculty of Mathematics and Physics, Charles University in Prague, Ke Karlovu 5, Prague 2, CZ-121 16 Czech Republic; 4 Department of Chemistry, University of Wyoming, 1000 E. University Ave, WY 82071 Laramie, USA; 5 Institute of Chemistry and Biochemistry, Faculty of Agronomy, Mendel University in Brno, Zemědělská 1, CZ-613 00 Czech Republic; 6 CEITEC-Central European Institute of Technology, Mendel University in Brno, Zemědělská 1, CZ-613 00 Brno Czech Republic; 7 Department of Molecular, Cellular, and Biomedical Sciences, University of New Hampshire, 46 College Road, Durham, NH 03824 USA; 8 ISCARE IVF a.s, Jankovcova 1692, CZ-160 00 Praha 6, Czech Republic; 9 VFN Gynekologicko-porodnická klinika, Apolinářská 18, CZ-120 00 Czech Republic

## Abstract

In this work, we shed new light on the highly debated issue of chromatin fragmentation in cryopreserved cells. Moreover, for the first time, we describe replicating cell-specific DNA damage and higher-order chromatin alterations after freezing and thawing. We identified DNA structural changes associated with the freeze-thaw process and correlated them with the viability of frozen and thawed cells. We simultaneously evaluated DNA defects and the higher-order chromatin structure of frozen and thawed cells with and without cryoprotectant treatment. We found that in replicating (S phase) cells, DNA was preferentially damaged by replication fork collapse, potentially leading to DNA double strand breaks (DSBs), which represent an important source of both genome instability and defects in epigenome maintenance. This induction of DNA defects by the freeze-thaw process was not prevented by any cryoprotectant studied. Both in replicating and non-replicating cells, freezing and thawing altered the chromatin structure in a cryoprotectant-dependent manner. Interestingly, cells with condensed chromatin, which was strongly stimulated by dimethyl sulfoxide (DMSO) prior to freezing had the highest rate of survival after thawing. Our results will facilitate the design of compounds and procedures to decrease injury to cryopreserved cells.

## Introduction

Application of cryopreservation to living cells and tissues has revolutionized biotechnology and modern medicine^1,2^. However, extensive damage occurs to a percentage of frozen and thawed cells and tissues. Though the freeze-thaw process can be greatly affected by the use of cryoprotective additives to improve cell viability^3,4^, the effects of freezing and cryoprotectants *per se* on the complex status of cell nuclei (and the genetic information contained therein) remain controversial^4–7^. Contradictory results in the literature have prevented a consensus on the fundamental question of the extent of DNA and chromatin fragmentation that occurs during freezing and thawing^8–11^. Moreover, even subtle changes to the chromatin structure can be expected to affect the viability and/or genetic information of freeze-thawed cells.

Concerning practical applications, it is very important to know which factors associated with freezing and thawing are responsible for the observed increase in the incidence of defects in live births resulting from *in vitro* fertilization^4,12–15^. Additionally, developments in the field of cryosurgery have the promise of positive therapeutic outcomes with few side effects in the treatment of certain cancers (e.g., skin, breast and liver)^16^. However, regarding the sensitivity of different cancer cells to low temperatures^17^, there is a lack of deep understanding of the mechanisms underlying this phenomenon as few studies have sought to compare the responses of normal somatic cells and cancer cells to freezing and thawing. Normal (non-transformed) cells largely differ in their resistance to freezing and thawing; for example, oocytes are extremely cryosensitive^18^. The condition and status of chromatin are critical for cell survival and functioning as well as for the preservation of unchanged genetic information. Therefore, varying sensitivities of chromatin to cryodamage may be an important factor as to why different cells respond differently to the freeze-thaw process. This topic, however, requires further exploration.

In our previous work^3^, we focused on the formation of ice during freezing as an important parameter that strongly influences cellular destruction and examined specific properties of selected cryoprotectant solutions during freezing, including dimethyl sulfoxide (DMSO), trehalose and a recombinant antifreeze fusion protein (AFP) that was originally isolated from the *Anatolica polita* desert beetle^2,3^. Building on this knowledge, here, we used these cryoprotectants to investigate the importance and extent of chromatin damage in freeze-thawed cells, specifically fragmentation and structural changes of chromatin. We described the post-freeze-thaw status of cells from two major perspectives: (i) the widely debated damage to DNA integrity, which can directly lead to death or genetic defects in cryopreserved cells, and (ii) the previously unexplored, less prominent alterations in the functional status of the higher-order chromatin structure and its impact on the viability of freeze-thawed cells.

In the present study, we correlate cell viability with freeze-thawed DNA integrity and chromatin states as explored by high-resolution confocal fluorescence microscopy and flow cytometry^19–23^, and we are the first to identify novel critical attributes of chromatin damage, shedding new light on the mechanisms of freeze-thaw-induced chromatin alteration, consequent cell survival, and cryoprotection. DNA double strand breaks (DSBs) represent the most serious DNA lesions^20,21,24,25^, but their induction through the freeze-thaw process remains controversial^26–29^. We have shown that freezing and thawing preferentially damage replicating (S-phase) cells by causing the collapse of replication forks, eventually leading to DSBs, thereby making rapidly dividing cells more sensitive to freeze damage. Excepting S-phase cells, in contrast to many earlier reports, we found that the freeze-thaw process does not directly induce DSBs; instead, it alters cells’ higher-order chromatin structure. The results of the present study, which was performed on normal human skin fibroblasts (NHDFs) and mammary carcinoma cells (MCF7s), significantly enhance our understanding of the freezing process and its impacts on normal and cancer cells, which can contribute to the future rational design of cryofunctional materials and cryotherapy. NHDF and MCF7 cells were chosen as they 1) allow to compare behavior of normal and cancer cells, 2) differ in higher-order chromatin architecture^2^ (which is relevant in the context of the present work) and 3) skin and breast cancers are often mentioned in literature as two of the preferred candidates for cryoablation^16^.

## Results

### DNA integrity, double-strand break induction, and replication fork collapse in cryopreserved cells

There is no consensus in the literature^6,30^ regarding DNA double strand break (DSB) induction (chromatin fragmentation) in cells that have undergone freezing and thawing. We identified a new type of DNA lesion that is associated with freezing and thawing, that specifically appears in replicating (S-phase) cells and that can be labelled with γH2AX and 53BP1 antibodies. Nuclear patterns of γH2AX/53BP1 foci in affected cells co-localize with collapsed replication forks, which can be converted to DSBs. We also found extensive alterations of higher-order chromatin structure, even in non-S-phase freeze-thawed cells. However, non-S-phase cells did not suffer from increased DSB induction.

**Figure 1:**
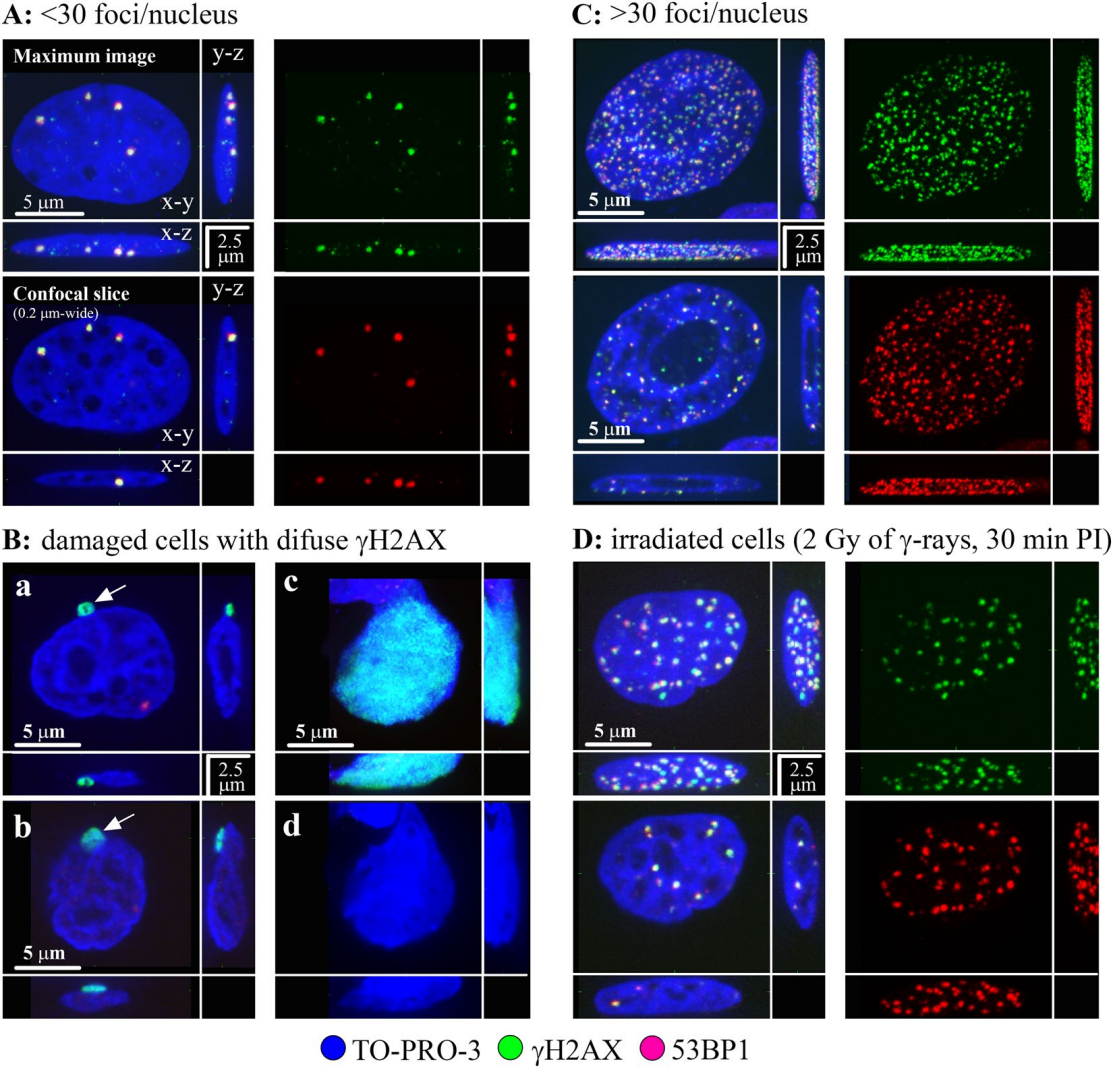
Three main categories (**A–C**) of MCF7 cells according to their γH2AX signal upon freezing/thawing. (**A**) The majority of cells remained unaffected by freezing/thawing in terms of DSB induction (category A, <30 γH2AX/53BP1 foci); the cells of this category were typical for DMSO-treated samples since in unprotected ones or those incubated with trehalose, a majority/substantial proportion of cells had damaged nuclei (**B**). In category (**B**) nuclei were stained with diffuse, localized (a,b) or pan-nuclear (c) γH2AX signals that did not colocalize with 53BP1. Cells with localized intense γH2AX signals largely preserved chromatin structure but with localized structure less chromatin protrusion(s) from the cell nucleus (white arrow). The chromatin structure of cells with pan-nuclear γH2AX was frequently altered (d), typically decondensed with complete loss of structure (panel d only shows chromatin staining). (**C**) A fraction of cells showed, for all cryoprotectant treatments, extremely high numbers of tiny γH2AX foci (green) that colocalized with 53BP1 protein foci (red) (category C, >30 γH2AX/53BP1 foci). The overall chromatin structure of these cells remained preserved, especially in cryopreserved samples. Preservation of higher-order chromatin structure for cells A and C is demonstrated in 3D confocal (0.3 μm-thick) images. (**D**) Formation of γH2AX/53BP1 foci at sites of DNA double strand breaks (DSBs) in MCF7 cells irradiated with 2 Gy of γ-rays (^60^Co, 1 Gy/min) and visualized 30 min post-irradiation. γH2AX – green, 53BP1 – red, TO-PRO-3 (chromatin) – blue.

**Figure 2:**
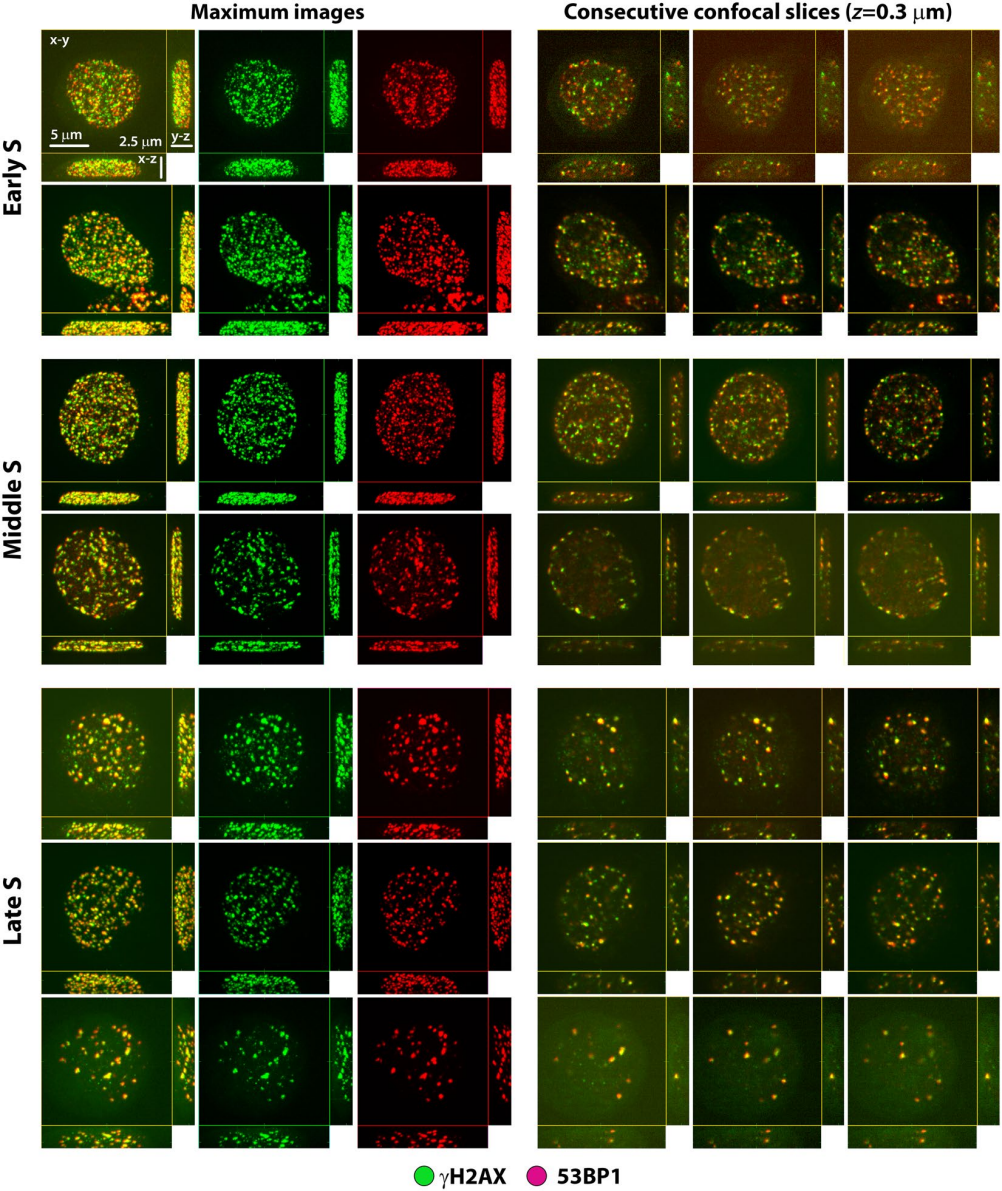
Three subcategories of MCF7 cells with increased numbers of γH2AX/53BP1 foci/nucleus after freezing/thawing, as determined using immunofluorescence confocal microscopy. Category 1 (top): nuclei with about >100 small γH2AX/53BP1 foci dispersed throughout the nucleus. Category 2 (middle): nuclei with >100 small γH2AX/53BP1 foci but distributed largely along the nuclear rim. Category 3 (bottom): nuclei with approximately 30 to 100 both small and large γH2AX/53BP1 foci distributed along the nucleolus and irregularly throughout the rest of the nucleus. There is evident similarity between the described γH2AX patterns (numbers and distribution) and the patterns of early (top), mid (middle), and late (bottom) S-phase replication (left captions). γH2AX foci (cells of category 1–3) thus seem to represent replication forks in S-phase cells collapsed upon freeze/thaw. 3D projections (x-y, x-z and y-z) of representative nuclei are shown for the composed of 40 confocal slices, each 0.3 μm thick (left column) and three consecutive single confocal slices, 0.3 μm thick (remaining columns). For maximum images, γH2AX (green) and 53BP1 (red) signals are also shown separately to demonstrate their mutual colocalization. Chromatin staining suppressed to improve the visibility of γH2AX/53BP1 foci.

Using high-resolution immunofluorescence confocal microscopy to detect γH2AX and 53BP1 signals^31^, which are the two accepted markers of DSBs, we compared DSB induction in frozen and thawed cells that had been cultured in standard medium (untreated cells) or in standard medium supplemented with cryoprotectants of different classes (AFP, trehalose, DMSO or trehalose + DMSO; Methods), which have different abilities to penetrate cells (or even the cell nuclei) and influence the chromatin status. Since γH2AX foci can be formed in the absence of DSBs^32^, in parallel, we co-stained cells with an additional DSB marker, the protein 53BP1^33^. We also irradiated MCF7 and NHDF cells with 1 Gy or 2 Gy of γ-rays as a DSB positive control. This process resulted in the rapid development of relatively large γH2AX foci co-localized with the 53BP1 repair protein 30 min post-irradiation, showing extensive formation of γH2AX/53BP1 (DSB) foci with approximately 23 DSB/Gy/nucleus (Figs 1 and 2 and S1).

**Figure 3:**
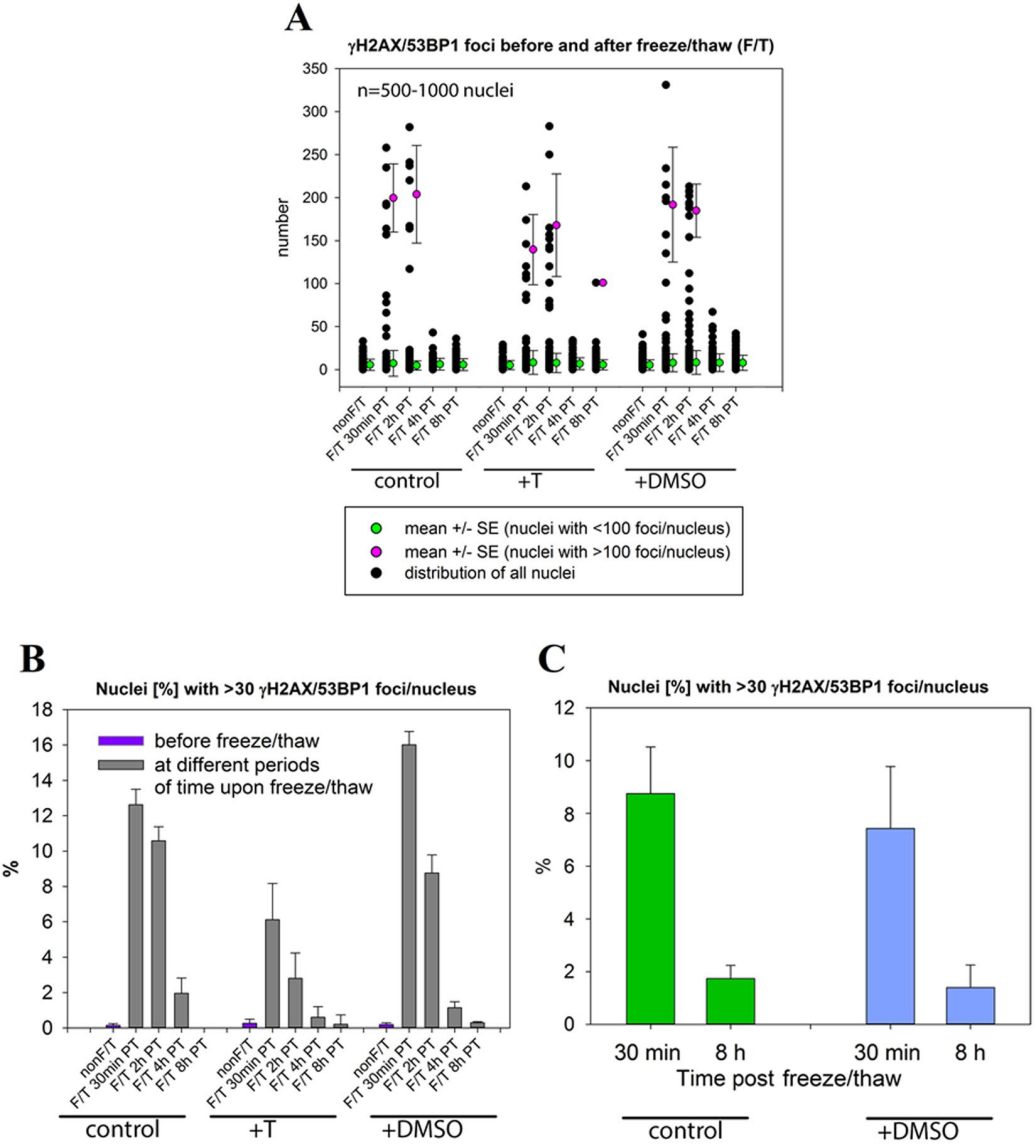
Distributions of γH2AX/53BP1 focus numbers per nucleus and proportions of cells with >30 γH2AX/53BP1 foci/nucleus in never frozen MCF7 cell samples and samples that were freeze-thawed with or without cryoprotectants. (**A**) Distributions of γH2AX/53BP1 focus numbers per nucleus before and in different periods of time after freezing/thawing. To emphasize presence of cells with “extreme” focus values (>100), the average numbers of γH2AX/53BP1 foci and their SDs were separately calculated for nuclei with <100 foci/nucleus (green circles) and >100 foci/nucleus (purple circles). For cells with <100 or <30 foci there is no statistically significant difference between the distributions of γH2AX/53BP1 foci/nucleus before and after freezing/thawing, irrespective of the cryoprotectant used (Kruskal-Wallis one way analysis of variance on ranks + Mann-Whitney rank sum test, p < 0.05 considered as significant). The fractions of cells with <100 or <30 foci/nucleus represented vastly dominant cell populations (i.e., approximately 93% or 86% of all cells), even in freeze-thawed cultures. Samples evaluated 30 min and 2 h post-freezing/thawing, which contain maximum numbers of cells with >100 foci/nucleus, were used for these calculations. Proportion [%] of MCF7 (**B**) and NHDF (**C**) cells with >30 γH2AX/53BP1 foci/nucleus determined prior to and in different periods of time after freezing/thawing. Results are compared for cells incubated or non-incubated with the indicated cryoprotectants. Horizontal axis – hours after the freeze/thaw cycle; N – never-frozen cells).

The cryoprotectants had minor effects on the viability of non-frozen cells (Table S1, Data 1, 2) and did not lead to increased DSB induction (Figs 3A and S1). As expected, the highest decrease in viability was found for trehalose or trehalose plus DMSO-treated (24 h incubation in trehalose + 5 min DMSO) MCF7 cells as they were also more sensitive to negative cryoprotectant viability effects than NHDF cells (Table S1, Data 1 and 2).

After freezing and thawing (−80 °C; rate of −1 °C/min), we identified important DNA defects that were associated with this process in both NHDF and MCF7 cells. A significant proportion (Table 1) of freeze-thawed cells exhibited a dramatically enhanced number of γH2AX/53BP1 foci (>30 to >100 foci/nucleus; Figs 1–3, S1). The nuclear patterns (number plus spatial distributions) of the γH2AX/53BP1 foci in cells with >30 to >100 γH2AX/53BP1 foci/nucleus were clearly different from the other freeze-damaged cells (Figs 1 and 2) and from cells exposed to γ-radiation (Figs 1 and S1). A careful inspection of images in 3D-space allowed us to sort cells with high numbers of γH2AX/53BP1 foci into three categories, as presented in Figs 2 and S2. Nuclei belonging to the first category contain approximately 100 or more small γH2AX/53BP1 foci dispersed throughout the nucleus. Nuclei in the second category have similar numbers of small γH2AX/53BP1 foci, but these foci are concentrated along the nuclear rim. Finally, nuclei in the third category show a mixture of 30 to 100 of both small and large γH2AX/53BP1 foci distributed along the nucleolus and irregularly throughout the rest of the nucleus. The γH2AX/53BP1 foci patterns in categories 1, 2 and 3 perfectly corresponded to those observed in early-, mid-, and late-S-phase (replicating) cells with collapsed (e.g., by camptothecin or topotecan) replication forks^34,35^. Our results thus show that the γH2AX foci (cells in categories 1–3) represent replication forks in S-phase cells that had collapsed upon freeze-thaw.

**Table 1:** Fractions [%] of NHDF and MCF7 cells with >30 γH2AX/53BP1 foci before freezing and after freezing/thawing compared to the fraction [%] of S-phase cells.

Treatment	NHDF cells				MCF7 cells			
	Non-frozen		Freeze/thawed (30 min after F/T)		Non-frozen		Freeze/thawed (30 min after F/T)	
	Cells with > 30 foci (CM)	S-phase cells (FC)	Cells with > 30 foci (CM)	S-phase cells (FC)	Cells with > 30 foci (CM)	S-phase cells (FC)	Cells with > 30 foci (CM)	S-phase cells (FC)
Untreated	0.5 ± 0.2	8.2 ± 2.5	9.0 ± 0.5	7.7 ± 2.5	0.5 ± 0.2	13.4 ± 0.2	13.8 ± 2.1	14.3 ± 0.4
Trehalose	0.5 ± 0.2	5.5 ± 1.5	3.8 ± 0.2	4.4 ± 0.6	0.5 ± 0.2	8.0 ± 0.5	6.2 ± 2.2	9.5 ± 0.6
DMSO	0.5 ± 0.2	6.9 ± 1.7	5.2 ± 0.4	8.3 ± 1.6	0.5 ± 0.2	14.7 ± 1.2	16.0 ± 2.0	15.8 ± 0.3
DMSO + trehalose	0.5 ± 0.2	5.3 ± 2.2	4.0 ± 0.2	5.3 ± 1.5	0.5 ± 0.2	7.3 ± 0.5	7.3 ± 2.3	10.2 ± 0.6

Results for the indicated cryoprotectant treatments are shown. The fractions of cells with >30 γH2AX/53BP1 foci were determined using immunofluorescence confocal microscopy (CM) prior to and 30 min after freezing/thawing. S-phase cells were quantified at the corresponding times by flow cytometry with propidium iodide (PI) staining. Values represent the means and standard errors.

**Figure 4:**
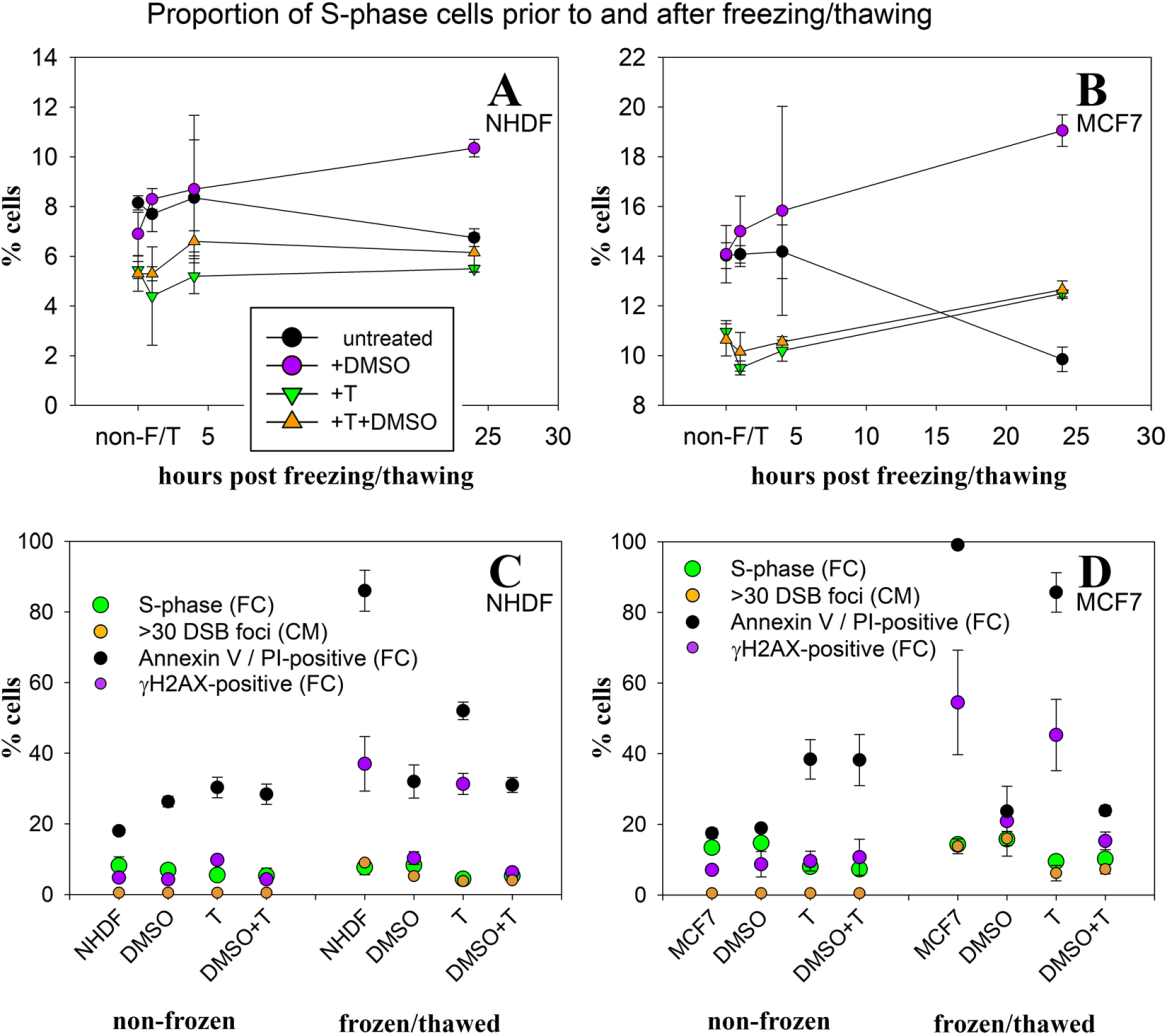
Fractions [%] of S-phase NHDF **(A)** and MCF7 **(B)** cells before freezing and at different time periods after freezing/thawing, determined by flow cytometry (PI staining), for the indicated cryoprotectant treatments. Dish-attached cells and cells released into culture media were included into the analyses. Fractions [%] of NHDF **(C)** and MCF7 **(D)** cells with >30 γH2AX/53BP1 foci, determined using immunofluorescence confocal microscopy (CM) prior to and 30 min after freezing/thawing (F/T), are compared to flow cytometric fractions of S-phase cells, γH2AX-positive cells and Annexin V/PI-positive cells quantified using flow cytometry (FC) prior to and 30 min after F/T. S-phase cells were identified using propidium iodide (PI) staining to show DNA content. Values represent the means and standard errors (see Table S1 for all the values).

For never frozen and freeze-thawed cells, we directly compared the proportions of cells with >30 γH2AX/53BP1 foci/nucleus with the proportions of replicating (S-phase) cells, as determined by flow cytometry (PI staining) (Table 1, Fig. 4, and Data 3, 4). The number of never frozen S-phase cells was higher for faster-propagating MCF7 cells (approximately 13%) than for NHDF fibroblasts (approximately 8%) and remained nearly the same before and immediately (30 min) after freezing and thawing (Table 1, Fig. 4). Importantly, the S-phase cell fractions corresponded to the percentage of cells with >30 γH2AX/53BP1 foci in freeze-thawed cell populations. By contrast, the fraction of cells containing more than 30 γH2AX/53BP1 foci/nucleus was very rare (<1%) among never frozen MCF7 and NHDF cells, either treated or untreated with cryoprotectants (Table 1, Fig. 4). The substantial fraction of freeze-thawed cells with >30 γH2AX/53BP1 foci per nucleus, therefore, likely corresponds to S-phase cells with collapsed replication forks.

**Figure 5:**
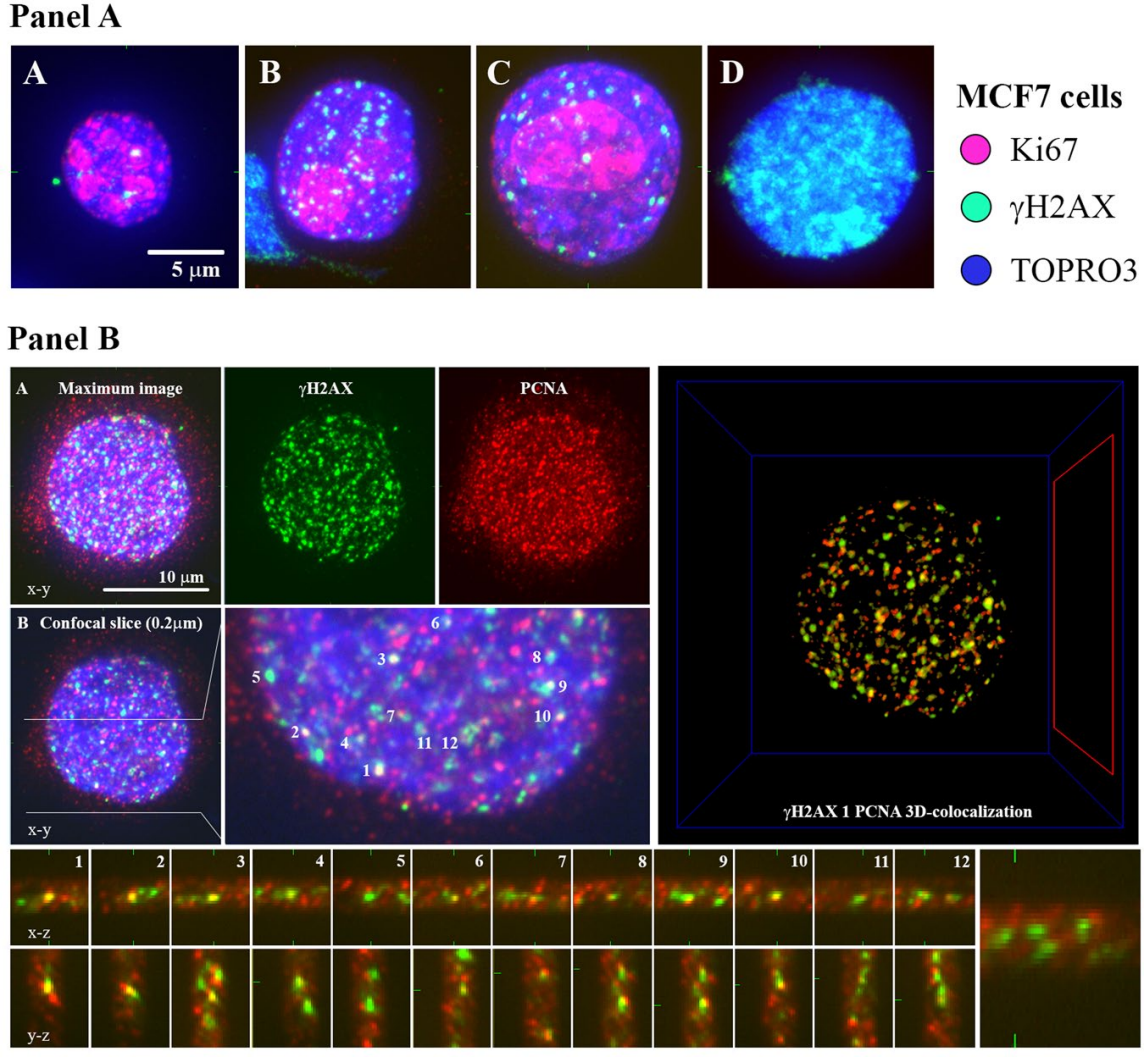
Cells with >30 γH2AX/53BP1 foci are S-phase cells with collapsed replication forks. Panel (A) Immunofluorescence microscopy of freeze-thawed MCF7 cells, stained with anti-γH2AX (green) and anti-Ki67 (red) antibodies, demonstrates the S-phase status of the cells with >30 γH2AX foci. (**A**) Cell nuclei with <30 γH2AX foci mostly showed jaguar-like patterns of Ki67 distribution typical of G1-phase or nucleolar staining with nucleoplasmic staining typical of G2-phase. (**B**,**C**) Cell nuclei with >30 γH2AX foci predominantly showed intense nucleolar Ki67-staining as typical for S-phase cells. (**D**) Most nuclei with pan-nuclear γH2AX staining (apoptotic or disintegrated nuclei) showed no Ki67 signal. Maximum projection images composed of approximately 40 confocal slices, each 0.3 μm thick, are displayed. Chromatin was counterstained with TO-PRO-3 (artificially colored blue). Panel (B) Immunofluorescence microscopy of frozen/thaw MCF7 cells, stained with anti-γH2AX (green) and PCNA (red) antibodies, demonstrates the colocalization of γH2AX foci with replication forks (PCNA) in cells containing >30 γH2AX foci. (**A**) Maximum projection images (composed of approximately 40 confocal slices, each 0.3 μm thick), showing patterns for anti-γH2AX and anti-PCNA antibodies, merged and separately. (**B**) Same as (A) but only showing the central confocal slice. The right panel displays an enlarged view of the colocalization of γH2AX and PCNA signals in the x-y plane. Detailed 3D colocalization for twelve γH2AX foci indicated in (panel B, bottom). Software-generated 3D-colocalization for all γH2AX and PCNA foci (panel B, right; Acquiarium software). Chromatin was counterstained (where relevant) with TO-PRO-3 (artificially colored blue).

The γH2AX foci in cells with >30 γH2AX/53BP1 foci per nucleus colocalized with 53BP1 (Figs 1, 2, and S2), and in a parallel experiment, they also colocalized with proliferating cell nuclear antigen (PCNA) (Fig. 5), a key factor in DNA replication that is localized in the replication fork. These results confirmed the identification of γH2AX/53BP1 foci with collapsed replication forks in cells with >30 γH2AX/53BP1 foci. The proliferative status of these cells was further supported by immunofluorescence microscopy images of freeze-thawed MCF7 cells that were stained with anti-γH2AX and anti-Ki67 antibodies, which revealed preferential nucleolar Ki67-staining, as is typical of S-phase, in the majority of the cells with >30 γH2AX foci (Fig. 5).

Cryoprotectants added to the cell cultures prior to freezing and thawing did not influence the susceptibility of cells to replication fork collapse (Fig. 3, Table S1). Only trehalose reduced the proportion of freeze-thawed cells with >30 γH2AX/53BP1 foci; however, this cryoprotectant exerted the same effect on the total number of S-phase cells, both in never frozen and freeze-thawed cells (Fig. 4).

Regardless of the cryoprotectant used, the number of cells exhibiting >30 γH2AX/53BP1 foci/nucleus decreased at 8 h after freezing and thawing (Fig. 3), indicating that a proportion of S-phase cells with collapsed replication forks either repaired their lesions (with potential consequences to genetic information due to misrepair) or died due to extensive damage (Fig. 4). During the post-thaw period, damaged S-phase cells were replaced by new cells entering the S-phase. In the post-thaw period, the portion of S-phase cells decreased only in the untreated control (Fig. 4). Unprotected cells, therefore, suffer from other (or additional, in case of S-phase cells) types of serious (chromatin) damage.

In addition to immunofluorescence microscopy (Fig. 4, Table S1), we quantified γH2AX-fluorescence using flow cytometry, which provided us with a broad view of chromatin defects in a statistically relevant number of cells. All freeze-thawed samples showed higher fractions of cells with increased γH2AX-fluorescence compared to never frozen cells, for both normal human skin fibroblasts and MCF7 mammary carcinoma cells. The highest increase (up to approximately 40%, 30 min after thawing) appeared in unprotected controls; these values were lower in trehalose-treated samples and were lowest in cells treated with DMSO or a combination of trehalose + DMSO. Flow cytograms of cells stained for γH2AX are shown in Data 5 and 6.

A comparison of the confocal microscopy and flow cytometry provided some interesting results. The relative number of S-phase cells with >30 γH2AX/53BP1 foci/nucleus was strongly correlated with the relative number of all γH2AX-positive cells, as determined by flow cytometry of cells frozen and thawed with DMSO (Fig. 4, Table S1). By contrast, the proportion of (damaged) replicating cells with >30 γH2AX/53BP1 foci was much lower than the proportion of all cells positive for γH2AX (by flow cytometry) in cells frozen and thawed with no cryoprotectant or with trehalose. In these cultures, microscopy revealed numerous cells with higher-order alterations in chromatin structure (TO-PRO-3 staining), chromatin protrusions (stained with γH2AX but not 53BP1) (Figs 1 and S3), or completely disintegrated chromatin, eventually manifested as pan-nuclear γH2AX fluorescence (Figs 1 and S4).

In summary, S-phase cells exhibited collapsed replication forks after freezing and thawing, which could not be prevented by any of the cryoprotectants studied. Collapsed replication forks thus represent the dominant type of damage in cryopreserved cells (DMSO or trehalose + DMSO), in which other effects of the freeze-thaw process on chromatin are minimized, as described in the following chapter. Hence, larger proportions of rapidly dividing cell populations, such as embryos and cancer cells, are more prone to freeze-thaw-induced DNA damage and eventual death compared to more slowly dividing cells^36–38^. However, a small fraction of damaged S-phase cells can survive the freeze-thaw process with collapsed replication forks. This condition, potentially more frequent in cancer cells for several reasons (e.g., higher proliferation, resistance to cell death, genomic instability), threatens the quality of the genome, even if these errors are not converted into DSBs^36,37,39^.

### Higher-order chromatin structure in cryopreserved cells

Next, we examined other important sources of defects that affect genetic information maintenance in cryopreserved cells – higher-order chromatin structure changes. The higher-order chromatin structure plays an important role in the regulation and maintenance of fundamental cellular processes, such as DNA transcription, replication and repair. It can also be considered to be a summarized representation of the epigenetic cell status. We compared chromatin texture (degradation of chromatin domains) and chromatin condensation before and after freezing and thawing (−80 °C; rate of −1 °C/min) in cells cultured in standard medium or in media containing cryoprotectants (AFP, DMSO, trehalose, and trehalose + DMSO; Methods).

**Figure 6:**
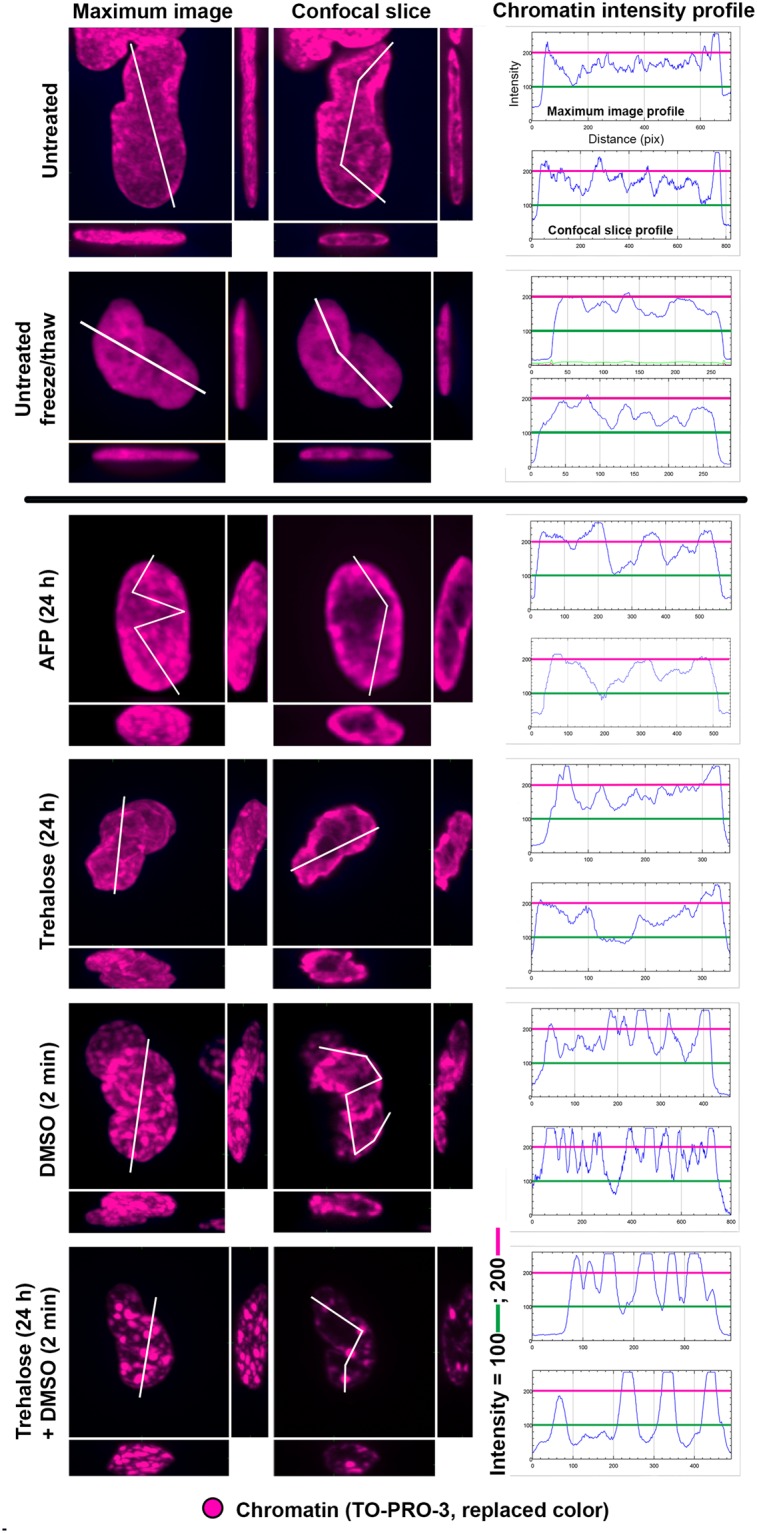
Effects of a freeze/thaw cycle on the higher-order chromatin structure of NHDF fibroblasts in the presence of cryoprotectants. Top row: untreated control cells that were not frozen. Other rows: cells frozen in the presence of the indicated cryoprotectant. ‘Maximum images’ are composed of 40 superimposed 0.3-μm thick confocal slices and are shown with x-z and y-z projections (left columns). The left columns present single confocal slices (0.3-μm thick) through the central nuclear plane with x-z and y-z projections. Chromatin was counterstained with TO-PRO-3 (blue or violet to better visualize the chromatin structure). Right column: intensity profiles of the violet (chromatin) channel along the demarcated paths (white lines) drawn above the maximum images and confocal slices of the cell nuclei. Each profile path was chosen to include nuclear areas with the maximum and minimum chromatin (violet) intensity. The intensity profiles show differences in the nuclear chromatin ‘texture’ and chromatin condensation; RFU [0–255] indicates relative fluorescence. Violet and green lines, which correspond to RFU = 100 and RFU = 200, respectively, are shown to facilitate comparisons. Images were acquired 30 min after thawing.

**Figure 7:**
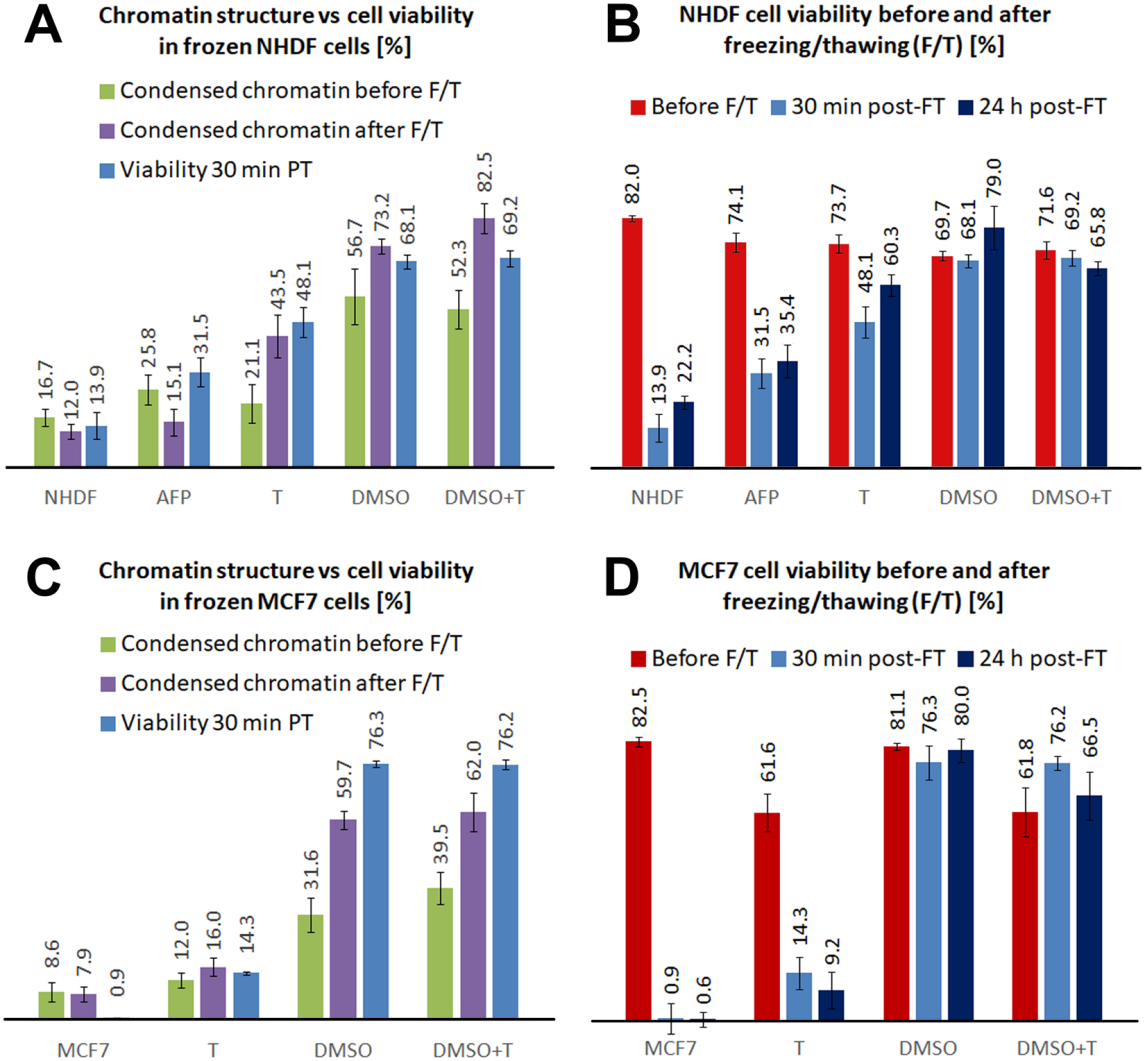
Effects of cryoprotectants on the chromatin structure of NHDF fibroblasts (**A**) and MCF7 cells (**C**) after a freeze/thaw (F/T) cycle. For freeze-thawed cells, the levels of chromatin condensation were correlated with the cryoprotective effects of cryoprotectants, quantified as the proportion of surviving (24 h after thawing) cells identified by flow cytometry (Annexin V/7-AAD staining). (**B**,**D**) Survival of NHDF fibroblasts (**B**) and MCF7 cells (**D**) compared for 30 min and 24 h after freezing/thawing. AFP was only used with fibroblasts due to the lack of this unique material.

To clarify the relationship between the chromatin state (Figs 6 and S5) and viability of cells upon freezing and thawing, we statistically quantified the changes in chromatin for each cryoprotectant and compared them with cell viability (Table S2, Fig. 7), as assessed by flow cytometry (Fig. S6, Data 1, 2). We performed these measurements at 30 min and 24 h (Table S2, Fig. 7) post-thaw to evaluate immediate cell damage resulting from freezing and thawing (disintegration, necrosis, etc.) and to potentially observe additional cell dying from delayed apoptosis, respectively. In the present study, all the viability values were obtained with a new software (Guava InCyte, Methods) allowing us more precise analyses. First, we used the same gates in all experiments, and second, in all cases, we excluded cell debris more precisely (as compared to RSC Adv.)^3^. The same gating and the same style of debris exclusion is extremely important to correctly evaluate viability of freeze-thawed cells because many of these cells are more or less fragmented. With the software used in RSC Adv^3^. (MUSE machine original software) this was not feasible.

First, we analyzed the effects of the cryoprotectants on never frozen cells (Fig. 7, Table S2). As quantified for NHDF fibroblasts, never frozen cells incubated with trehalose or AFP exhibited an uninfluenced chromatin structure with the proportion of cells with condensed nuclear chromatin (~20–25%) similar to that of the untreated control, i.e., never frozen cells without cryoprotectants (~17%).

By contrast, application of DMSO to never frozen NHDF cells caused extensive chromatin condensation in approximately 57% of cells, which was a 40% increase compared to the control.

The quantitative effects of freezing and thawing on the nuclei of untreated and cryoprotectant-treated NHDF and MCF7 cells are summarized in Fig. 7 and Table S2. Freezing and thawing of untreated cells caused serious chromatin damage (largely decondensed, disrupted chromatin structure) that was detrimental to cell survival–roughly 14% of untreated cells survived the freeze-thaw process (Figs S6 and S7 Data 1, 2).

To objectively quantify the level and statistical occurrence of chromatin structure and condensation among the observed cell nuclei, in addition to performing visual inspections of chromatin in large sets of cells, we calculated a 2D Fourier transform (Suppl. Info.) in polar coordinates from a single (central) 2D section of the 3D image z-stack. To discriminate nuclei with condensed chromatin, the best criterion appeared to be the first momentum of the middle frequency of the Fourier-transformed data component (Fig. S8). The discrimination criterion was 7.5×10^–6^ (where unity is the zero frequency maximum). Typical trehalose- and AFP-treated condensed chromatin cells were close to the boundary defined by the first momentum of the middle frequency of the Fourier-transformed data between condensed and standard or disintegrated chromatin, while typical DMSO-treated cells were farther from this condensation border.

Cryoprotectants had a significant effect on the chromatin structure and cell survival upon freezing and thawing (Fig. 7, Table S2). First, we describe the results for NHDF fibroblasts. AFP exerted the smallest effect on chromatin condensation and NHDF cell survival (approximately 35%) among the cryoprotectants studied. Due to its large molecular size, the effect of AFP is restricted to the extracellular space, where it binds to ice crystals, thus mitigating cell damage caused during the freezing process. AFP was studied only with NHDF fibroblasts due to a lack of this unique material.

Regarding DMSO, the freeze-thaw process further increased chromatin condensation that was already induced by this compound prior to freezing. The proportion of cells with condensed chromatin was 73% of DMSO-treated cells and increased to 83% of cells when DMSO was applied in combination with trehalose. Importantly, these values correlated well with the viability (i.e., the annexin V/PI negativity) of cells 30 min and 24 h after thawing. Approximately 68% of DMSO-treated cells survived freezing and thawing, while only 14% of untreated cells survived.

The high cryoprotective efficiency of DMSO could be, to a large extent, attributed to the ability of the small DMSO molecules to penetrate into cells and nuclei. Therefore, unlike many other cryoprotectants, DMSO can affect the freeze-thaw process directly in the cell nucleus and efficiently protect this vital organelle and chromatin (Table S2).

Finally, trehalose had an intermediate effect on freeze-thawed NHDF cells, inducing chromatin condensation in 44% of cells. This value was again close to the 30 min post-thaw cell survival rate of 48% in trehalose-treated cells. Importantly, when trehalose was applied for shorter periods of time (4 or 6 h), the freeze-thawed cell survival remained practically unaffected. The survival of NHDF cells was improved only when trehalose was applied for 24 h. This observation indicates that trehalose penetrates cells, but does so slowly. To further support this hypothesis, we analyzed the phenomenon using several mass spectrometry techniques (LC-MS/MS, DART and DESI). The LC-MS/MS results, which demonstrate and quantify the presence of trehalose in the filtered lysates of both cell types under study, are summarized in Fig. S9. DART successfully confirmed the identity of the trehalose peaks detected by LC-MS/MS. We further demonstrated the penetration of trehalose into intact cells using DESI with NHDF fibroblasts and two of the trehalose concentrations in the culture medium, as illustrated in Fig. S10. Trehalose in the cell cytoplasm affects the osmolarity in different cellular subcompartments, such as the nucleus, and therefore also affects the chromatin structure, which is in contrast to the solely extracellular effect of AFP.

In parallel, we performed the same set of experiments with MCF7 cells (Fig. 7, Table S2). Untreated MCF7 cells showed chromatin condensation at approximately half the rate of untreated NHDF fibroblasts (8.6% vs. 16.7%, respectively) before freezing and thawing. In accordance with the results for NHDF fibroblasts, only DMSO induced chromatin condensation in never frozen MCF7 cells. Among freeze-thawed MCF7 cells, we found about 60% of DMSO-treated cells and less than 8% of untreated cells had condensed chromatin. According to the annexin V/PI positivity results (Fig. 7 and Table S2), approximately 76% and 80% of DMSO-treated MCF7 cells survived for 30 min and 24 hours, respectively, after freezing and thawing, which markedly contrasted with the less than 1% survival rate of untreated MCF7 cells. Trehalose exerted an insignificant effect on chromatin condensation in MCF7 cells upon freezing and thawing. Chromatin condensation in these cells appeared only in ~16% of cells (44% in NHDF cells) and was less visually obvious than in NHDF fibroblasts treated with the same cryoprotectant (compare Figs 6 and S5). According to the annexin V/PI positivity results, trehalose-treated MCF7 cells had 14% survival at 30 min after freezing and thawing, which slightly decreased 24 hours later (Fig. 7). Unlike in NHDF fibroblasts, the combined treatment of DMSO and trehalose did not further increase either chromatin condensation (62%) (Fig. 7) or cell survival (76% and 67% at 30 min and 24 h post-thaw, respectively) (Figs S6 and S7) compared with the DMSO-only treatment (Table S2). MCF7 cell nuclei condensation upon cell incubation with cryoprotectants (studied before and after freeze/thaw) is shown in Fig. S11. DMSO was the treatment leading to highest nucleus condensation and smallest damage.

In contrast to surviving cells that had condensed chromatin, cells with decondensed chromatin were frequently stained, locally or pan-nuclearly, with a diffuse γH2AX fluorescence that was not colocalized with 53BP1 signal (Fig. 1) and indicated cell death. Cells with this type of γH2AX signal increased in number with the post-thawing time, except for the DMSO-treated sample, which had a consistently low percentage of these cells (Fig. S4). To evaluate the effects of chromatin condensation on thawed cell survival from the other effects of the cryoprotectants, we investigated the impact of a short pre-freezing incubation on the higher-order chromatin structure and cell viability using cells in media with different osmolarities (hypertonic, hypotonic and isotonic) (Fig. S12). Freeze-thawed cells treated with hypertonic medium showed the strongest chromatin condensation and the highest (approximately 40%) cell viability compared to the other osmolarities. On the other hand, the hypotonic pretreatment of non-frozen cells induced slight chromatin hypocondensation compared to the isotonic pretreatment. Upon freezing and thawing, the viability of hypotonic-treated cells was similar to that (below 10%) of cells frozen and thawed in normal (isotonic) medium. This further supports our assumption that chromatin condensation plays a positive role in improving cell survival during cryopreservation.

We conclude that the viability of NHDF and MCF7 cells upon freezing and thawing is strongly positively correlated with condensation of chromatin structure. Chromatin condensation is influenced by cryoprotective compounds to varying extents.

Altogether, non-replicating and replicating (S-phase) cells both suffer from non-DSB chromatin damage, i.e., more or less obvious changes in the higher-order chromatin structure, which can be efficiently reduced by DMSO and, to a lesser extent, by trehalose. Viability measurements at later periods of time after freezing and thawing (24 h) support the inference that chromatin condensation, provoked by cryoprotectants and protective of cells during freezing and thawing, does not negatively influence cell survival over the long term. S-phase cells, in addition, face replication fork collapse, potentially leading to the induction of multiple DSBs. However, further study is required regarding the influence of both DSB damage and non-DSB chromatin damage on genetic and epigenetic information.

## Discussion

Chromatin represents a critical target for cryodamage and cryoprotection. Recent progress in our understanding of chromatin biology has revealed that this material not only preserves genetic information but also plays important roles in the regulation and maintenance of fundamental cellular processes, such as DNA transcription, replication and repair. In this respect, the chromatin structure is of the utmost importance since it represents a unified manifestation of the epigenetic cell status and directly or indirectly influences the interactions of DNA and proteins. The status of cryopreserved freeze-thawed cells can be evaluated from two basic viewpoints: a) susceptibility of chromatin to the induction of DSBs, which can lead to genetic defects in freeze-thawed cells or to chromatin fragmentation (cell death), and b) susceptibility of chromatin to (epigenetic) alterations of its structure, with potential consequences on the expression of genetic information.

The relationship between an altered chromatin structure and carcinogenesis has been described in our earlier work, which revealed the presence of alterations in the higher-order chromatin structure in histologically normal tissue that was adjacent to the tumor^40^. Additionally, Leffak^41^ linked the amplification of repetitive sequences, which is the mechanism responsible for the development of numerous neurodegenerative diseases, to the repair of collapsed replication forks.

In the present study, we shed new light on the highly debated chromatin fragmentation of cryopreserved cells. Rather than the expected extensive induction of DSBs in the majority of cells undergoing the freeze-thaw processes^11,29^, we identified alterations in the higher-order chromatin structure, the extent of which was cryoprotectant-dependent. Moreover, for the first time, we described a new type of chromatin damage associated with freezing and thawing that is specific to S-phase cells and could not be mitigated by any of the cryoprotectants used in this study. The damage consists of extensive collapse of the replication forks, which could be potentially converted into DSBs^42–44^. Taken together, these findings may help explain why some studies have identified extensive chromatin disintegration upon freezing and thawing^11^ while others report only conditional chromatin fragmentation (e.g., in defective sperm)^7^ or the complete absence of DSB formation^8,9,45^ (reviewed in^5,10^). Furthermore, by identifying new fundamental attributes of cryodamage in NHDF and MCF7 cells, we describe important implications for cell cryoprotection and cryoablation.

With regard to DNA double strand breaks, we found that only approximately 14% of MCF7 and 9% of NDHF freeze-thawed cells exhibited an enhanced number of γH2AX/53BP1 foci, which is in contrast to numerous studies^42–44,46–48^. Importantly, these cells contained a minimum of 30 γH2AX/53BP1 foci/nucleus, but they typically had more than 100. The pattern of γH2AX/53BP1 foci in freeze-thawed cell nuclei corresponds to the patterns of the replication sites in S-phase cells, suggesting that cells with >30 γH2AX/53BP1 foci per nucleus represent replicating (S-phase) cells with collapsed replication forks, potentially leading to DSBs and genetic defects, as previously mentioned. The S-phase status of affected cells was further confirmed by the Ki67 patterns that are characteristic of the S-phase that were expressed by a majority of these cells and, more specifically, by the colocalization of γH2AX foci with the PCNA antigen (localized to replication sites). The total size of the S-phase subpopulations did not significantly change before and after the freeze-thaw process. Notably, MCF7 and NHDF cells that had >30 γH2AX/53BP1 foci/nucleus before freezing and thawing represented less than 0.5% of cells. These results suggest that S-phase cells are intact before freezing, but almost all experience a massive collapse of replication forks when they are freeze-thawed. Localization of 53BP1 to sites of stalled or broken replication forks, as observed in the present study, has been previously reported^49^.

The proportion of freeze-thawed NHDF and MCF7 cells with >30 γH2AX/53BP1 foci/nucleus was analyzed for various cryoprotectant treatments. DMSO treatment did not have a significant effect either on the proportion of total S-phase cells or on the number of S-phase cells with >30 γH2AX/53BP1 foci/nucleus. By contrast, in agreement with previous studies^50,51^, long (24 h) trehalose treatment somewhat inhibited cell division, i.e. decreased the total fraction of S-phase cells compared with that of untreated or DMSO-cryopreserved NHDF and MCF7 cell samples, even prior to freezing. To a comparable extent, trehalose also reduced the number of cells with >30 γH2AX/53BP1 foci/nucleus after freezing and thawing. This observation further confirms that cells with >30 γH2AX/53BP1 foci/nucleus were in the S-phase and suggests that the collapse of replication forks during freezing and thawing could not be prevented by any of the cryoprotectants studied.

The reduced incidence of cells with >30 γH2AX/53BP1 foci/nucleus among normal NHDF fibroblasts after the freeze-thaw process likely reflects their lower replication activity relative to cancerous MCF7 cells. As freezing and thawing preferentially and more seriously damages S-phase cells, rapidly dividing cells, such as tumors and embryos, are likely to be more sensitive to DNA cryodamage. To the best of our knowledge, this finding has not yet been reported. Replication-induced DSBs may extensively accumulate especially in cancer cells that frequently exhibit defects in DNA repair, particularly in homologous recombination^42,52^.

Next, we investigated changes in the epigenetic chromatin structure as another potential form of DNA damage in NHDF and MCF7 cells that had undergone a freeze-thaw cycle. Cells in standard culture medium with no added cryoprotectants exhibited chromatin structure alterations similar to cells in hypotonic medium when exposed to a freeze-thaw cycle. The majority of chromatin decondensed to varying extents, which frequently correlated with damage of the nuclear envelope (preliminary results). By contrast, DMSO stimulated chromatin condensation in never frozen NHDF and MCF7 cells, and chromatin remained condensed after thawing. Accordingly, cells of both types incubated with DMSO had the highest viabilities after thawing. The lower tendency of MCF7 cells to form condensed chromatin compared to NHDF fibroblasts may be explained by the differing levels of native chromatin condensation in untreated, never frozen NHDF and MCF7 cells^2^. It should be noted that chromatin condensation naturally appears in prometaphase cells along with mitotic chromosomes formation. Hence, unwanted cell synchronization induced by freeze/thaw processes or the cryoprotectants *per se* can theoretically increase the fraction of prometaphase cells with highly condensed chromatin. Nevertheless, except of the already described decrease of S-phase cells, we did not recognize any significant change in the cell cycle distribution after freezing/thawing. Moreover, our measurements of chromatin condensation were performed at 30 minutes after thawing, which is, in any case, very short period of time to experience manifestation of any alterations of the cell cycle. Therefore, chromatin condensation provoked in the present study by freezing/thawing can be attributed to cryoprotectant- and freeze/thaw-invoked processes independent of the cell cycle, as described below.

AFP and trehalose had only minor impacts on chromatin condensation prior to freezing and thawing. Trehalose molecules do not penetrate into the nucleus, and large AFP molecules protect cells only from the extracellular space^53^. Compared to the DMSO treatment, lower amounts of AFP- and trehalose-treated freeze-thawed cells had condensed chromatin and survived the freeze-thaw cycle. These results thus suggest that chromatin condensation is very important for protecting cells against damage during the freeze-thaw processes.

To test the importance of chromatin condensation on the viability of freeze-thawed cells separately from any additional effects of cryoprotectants (e.g., nuclear envelope stabilization, ROS sequestration, etc.), we treated NHDF cells with hypertonic and hypotonic media prior to freezing and thawing. The hypertonic treatment, which condensed chromatin, significantly reduced the adverse effects of freezing and thawing on NHDF cells compared to the hypotonic treatment, which decondensed chromatin. Moreover, cryoprotectant-induced chromatin condensation that protected cells during freezing and thawing did not have negative effects on the long-term (24 h) survival of these cells. Based on these results, we propose that chromatin condensation provoked prior to freezing is less destructive to higher-order chromatin structure than the abrupt condensation that takes place as a consequence of cellular dehydration during freezing. The condensed chromatin state reduces damage to the chromatin structure during freezing and prevents the loosening and dispersion of chromatin during the hypotonic shock induced by cell rehydration after thawing. Further chromatin condensation that occurs as a consequence of freezing *per se* is related to dehydration of the nucleus and chromatin, which leads to an increase in the intracellular concentration of ions. This condition, in turn, likely further stabilizes the condensed chromatin state and higher-order chromatin structure during freezing and thawing. Since condensed chromatin is largely surrounded by neighboring chromatin molecules and chromatin-bound proteins, it is better protected from the destructive effects of ice formation, and thus its integrity is preserved. Nevertheless, the possible effects of chromatin structure changes on the epigenetic cell status remain to be determined.

Based on these results, we propose two hypotheses as a possible mechanism of freeze-thaw-induced replication fork collapse: 1. The freeze-thaw process generates large amounts of reactive oxygen species (ROS) that extensively induce single-strand DNA breaks^54^. When the replication fork encounters this lesion, its progression is stopped and the fork may collapse and eventually be converted into a DSB. 2. Replication forks, which are sensitive to mechanical stress, may also be directly damaged by chromatin structure changes provoked by the freeze-thaw cycle. This latter explanation appears to be more probable (or more important) since DMSO, an efficient ROS scavenger^55^, does not reduce the number of collapsed replication forks. Indeed, in some experiments, the number of γH2AX/53BP1 foci that were colocalized with replication forks was higher in samples freeze-thawed with DMSO than in unprotected controls. This may suggest that chromatin condensation, provoked by the freeze-thaw process and further stimulated by DMSO, is harmful to the replication forks.

The literature provides differing conclusions regarding cell survival upon extensive replication fork damage^42–44,46–48^; therefore, it is difficult to predict the fate of freeze-thawed cells affected by this damage, particularly if the cells suffer from additional types of chromatin alterations. For instance, Dixon and colleagues described^56^ impaired DNA damage signaling and repair in cells exposed to hyperosmolar microenvironments, which caused chromatin condensation. Chromatin condensation, as observed in the present study after cell freezing and thawing, can therefore potentially decrease the efficiency of collapsed replication fork repair and increase the induction of mutations or cell death. In any case, it is reasonable to propose that a fraction of damaged S-phase cells can survive the freeze-thaw cycle, even with collapsed replication forks. Current evidence suggests that multiple mechanisms are used to repair replication damage, yet these mechanisms can have deleterious consequences on genome integrity^57^. This worsens the condition of the genetic information, even if these aberrations are not converted into DSBs. Mechanisms of how stalling DNA replication forks can promote either epigenetic or genetic changes were recently reviewed by Rowlands *et al*.^58^ and Leffak^41^, respectively.

In summary, the present study is the first to show that replicating (S-phase) cells are strongly affected by the collapse of replication forks during freezing and thawing. These collapsed forks may develop into DSBs. Rapidly dividing cell populations, such as tumors and embryos, may thus be more sensitive to this type of damage. The collapse of replication forks in S-phase cells was not prevented by any cryoprotectant studied. In addition to replication fork collapse, both replicating and non-replicating cells suffered from non-DSB chromatin damage, i.e., freeze-thaw-induced alterations of the chromatin structure and/or secondary chromatin fragmentation in cells with disintegrated or apoptotic nuclei, which could be efficiently reduced by DMSO and, to a lesser extent, by trehalose. Cells with strongly condensed chromatin prior to freezing had the highest rate of survival after freezing.

Cryoprotectants added to the culture media before freezing and thawing stabilized the nuclear integrity to varying extents and ensured sufficient cell amounts surviving the freeze-thaw process, but none of these factors protected S-phase cells from freeze-thaw-induced collapse of the replication forks.

Both collapsed replication forks and epigenetic structural chromatin damage may affect genetic information^39,59^ and can lead to defects in live births after *in vitro* fertilization. Taken together, these results provide a deeper understanding of the freezing process in cells, particularly its effect on cell nuclei, revealing potential risks associated with cell freezing but also facilitating the rational design of cryofunctional compounds and cryoprotective procedures.

## Methods and Materials

### Cells and cell culture

Certified normal human foreskin fibroblasts (NHDF) and human breast adenocarcinoma cells (MCF7) were obtained from CLS Cell Lines Service GmbH (Eppelheim, Germany), cultivated in DMEM medium (PAN Biotech, Aidenbach, Germany, cat. no: P03–0710), supplemented with 10% fetal calf serum (PAA Laboratories GmbH, Pasching, Austria) and 1% penicillin + streptomycin (stock solution mixture 10 000 U/mL penicillin + 10 mg/mL streptomycin; PAN Biotech, cat. no.: P06–07100), at 37 °C in a humidified atmosphere of 5% CO_2_. The cells obtained (at passage 2) were freshly defrost, multiplied, and used for experiments in low passages (about 5–7) to prevent possible effects of senescence on chromatin structure and cell functions.

### Cryoprotectants and cells freezing

Human fibroblasts and MCF7 cells were frozen down to −80 °C using a gradient of −1 °C/min in culture media (unprotected controls) or in culture media supplemented with particular cryoprotectant. The following concentrations of cryoprotectants were used in indicated concentrations: DMSO −10% (w/w), trehalose −3.2% (w/w) (i.e. 100 mM), DMSO + trehalose −10% (w/w) +3.2% (w/w) (i.e. 100 mM), and AFP (TrxA-ApAFP752) −0.5 mg.ml^−1^. D-(+)-trehalose dihydrate and DMSO were purchased from Sigma-Aldrich, AFP protein was prepared as described below. Cryoprotectants were injected to culture media of cryopreserved cells 24 h (AFP, trehalose) or 2 min (DMSO) prior to freezing. To prevent cytotoxicity of DMSO, DMSO-containing samples (DMSO and DMSO + trehalose) were cooled to 4 °C before adding DMSO to cells; also the remaining samples were precooled in the same way and then submitted to the freezing procedure. For the indicated treatments, the cryoprotectants had only minor effects on the viability of non-frozen cells as described in Results.

### Expression and purification of recombinant TrxA-ApAFP752 fusion protein

The recombinant plasmid pET32b-*Apafp752* was transformed into *Escherichia coli* Rosetta-gami 2(DE3) or BL21 (DE3)pLysS competent cells (Novagen). A single transformed colony was used to inoculate 25 mL of Luria-Bertani (LB) medium containing 0.3 mM ampicillin and cultured overnight at 37 °C and shaken at 225 rpm. 4 mL of the overnight culture was transferred into 1 L of fresh LB medium with 0.3 mM ampicillin and grown at 37 °C and 225 rpm until the optical density at 600 nm (OD_600_) reached 0.6–0.7. The culture was induced with 1 mL of 400 mM isopropanol-1-thio-β-D-galactopyranoside (IPTG), to cause over-expression of TrxA-ApAFP752, for 8 hours at 25 °C. The cells were harvested by centrifugation (20 minutes, 8700 g, 4 °C) and stored at −80 °C until purification.

The cell pellet was resuspended with ice-cold binding buffer (EDTA-free Halt™ protease inhibitor cocktail (Fisher), 50 mM sodium phosphate, 150 mM NaCl, 20 mM imidazole and Benzonase nuclease (Millipore) at pH 8.0) and lysed using a French press (1300 psi). The lysate was collected and centrifuged (20 minutes, 27200 g, 4 °C) to remove the cell debris. The supernatant was filtered through a 0.22 μm syringe-driven filter (Millipore) and concentrated to 5 mL before being purified by Fast Protein Liquid Chromatography (FPLC; GE Healthcare ÄKTA purifier 900) equipped with a HisTrap HP Ni-NTA column (GE Healthcare). The sample was loaded into the Ni-NTA column, washed with washing buffer (50 mM sodium phosphate, 150 mM NaCl, 20 mM imidazole at pH 8.0), and eluted with the same buffer containing 500 mM imidazole through a gradient elution. The fractions were pooled and dialysed in sodium phosphate buffer (50 mM sodium phosphate, 150 mM NaCl at pH 8.0) for 2 hours, then switched to fresh buffer and left overnight. The purity of the sample was analysed by 12.5% SDS-PAGE and stained with Coomassie blue; TrxA-ApAFP752 has a molecular weight of 27 kDa and was compared to a stained protein ladder (BioRad). Additional purification steps were taken to obtain a pure sample and SDS-PAGE was used after each step to analyse the purity. The sample was divided into three fractions that were each concentrated to 1 mL and run through a Superdex 75 10/300 GL size exclusion column (GE Healthcare) equilibrated with sodium phosphate buffer. Fractions that were still not pure were run through a Ni-NTA column and dialysed for a second time. Once the desired purity was obtained, the sample was buffer exchanged into a K_x_H_Y_PO_4_ buffer (50 mM potassium phosphate, 20 mM NaCl, and 1 mM NaN_3_ at pH 8.0). The concentration was estimated using UV-Visible spectrophotometry at 280 nm with the calculated extinction coefficient of 19,575 M^−1^cm^−1^. The sample was concentrated to 1 mL and lyophilized overnight. For further experiments, the protein was rehydrated as needed and buffer exchanged.

### Irradiation

Cells were irradiated in the culture medium at 37 °C from a ^60^Co source (Chisostat, Chirana, Prague, Czech Republic) with a dose of 1 Gy or 2 Gy (γH2AX/53BP1 foci immunofluorescence) or 5 Gy (flow cytometry); the dose rate was 1.0 Gy/min. During the irradiation, the cells were kept in a thermostable box, ensuring a constant temperature and prevention from infection during the whole procedure. After irradiation, the cells were immediately placed back into the incubator (37 °C/5% CO_2_) until the fixation.

### γH2AX phosphorylation by flow cytometry

Flow cytometry was used to quantify γH2AX phosphorylation in cells that were frozen with or without cryoprotectants. The Muse Cell Analyser (Merck Millipore) and Muse H2A.X Activation Dual Detection Kit (MCH200101, Merck Millipore), which discriminate between H2AX (−)/γH2AX (−) vs. H2AX (+)/γH2AX (−) vs. H2AX (−)/γH2AX (+) and H2AX (+)/γH2AX (+) cells, were used according to the manufacturer’s instructions. The cell state was analysed 1 h and 4 h after the cells were thawed.

### Cell cycle by flow cytometry

The Muse Cell Analyser (Merck Millipore) and Muse Cell Cycle Assay Kit (MCH100106) were used according to the manufacturer’s instructions (download at http://www.merckmillipore.com/CZ/cs/product/Muse-Cell-Cycle-Assay-Kit,MM_NF-MCH100106#anchor_DS). In the present study, all the viability values were obtained with new software (Guava InCyte soft. 3.1.1., Millipore) allowing us more precise analyses. We used the same gates in all experiments and, in all cases, we excluded more precisely cell debris (compared to RSC Adv.)^3^. The same gating and the same style of debris exclusion is extremely important to correctly evaluate viability of freeze-thawed cells because many of these cells are more or less fragmented. With the software used in RSC Adv^3^. (MUSE machine original software) this was not possible.

### Immunostaining

DSBs were quantified by high-resolution confocal microscopy as foci showing dual co-localization of γH2AX and 53BP1; together, these proteins are generally accepted as DSB markers^14^. The proteins were visualized using a mouse monoclonal antibody against H2AX phosphorylated at serine 139 (γH2AX) (dilution 1:500; Upstate) and with a rabbit polyclonal antibody against 53BP1 (dilution 1:500; Upstate). The maximum DSB signals typically appeared about 30 min post-DSB induction^21^. Hence, after complete thawing, the cells were maintained for an additional 30 min in the incubator (37 °C, 5% CO_2_) in fresh pre-heated (37 °C) DMEM medium supplemented with 10% FCS to allow full development of the foci. Secondary antibodies, namely, affinity-purified donkey anti-mouse-FITC-conjugated antibody (dilution 1:100) and affinity-purified donkey anti-rabbit-Cy3-conjugated antibody (dilution 1:200) (ImmunoResearch Laboratories, West Grove, PA), were used to visualize the primary antibodies. The immunostaining procedure was performed as described in *et al*.^19^ and Sevcik *et al*.^60^ with minor modifications. The nuclear envelope (Fig. S5) was stained with anti-lamin A/C monoclonal mouse antibody (dilution 1:1000; Sigma-Aldrich). Nuclear chromatin was counterstained with 1 μM TO-PRO-3 (Molecular Probes, Eugene, USA) in 2 × saline sodium citrate (SSC) prepared fresh from a stock solution. After the cells were briefly washed in 2 × SSC, the samples were mounted using Vectashield medium (Vector Laboratories, Burlingame, CA, USA).

### Cell survival (flow cytometry and Trypan blue exclusion assay)

Flow cytometry was used to quantify survival and apoptosis in cells that were frozen with or without cryoprotectants. The Muse Cell Analyser (Merck Millipore) and Muse Annexin V & Dead Cell Assay Kit (MCH100105, Merck Millipore), which discriminate between Annexin V (+)/Propidium Iodide (+) (live) vs. Annexin V (+)/Propidium Iodide (−) vs. Annexin V (−)/Propidium Iodide (+) and Annexin V (−)/Propidium Iodide (−) cells, were used according to the manufacturer’s instructions. The cell state was analysed 30 min or 24 h after the cells were thawed. Trypan blue exclusion assay was used according to standard protocol as an alternative method to determine the cell survival. TC20 Automated Cell Counter was used.

### Confocal microscopy and data analysis

Effects of the treatments were directly analysed on the microscopic slides under the microscope (about 500–2000 cells) and/or on acquired 3D images (>50 cells) (below). Two replicas of microscopic slides for each sample were directly evaluated by two experienced scientists. The following equipment was used for confocal microscopy and image acquisition: an automated Leica DM RXA fluorescence microscope (Leica, Wetzlar, Germany) equipped with an oil immersion Plan Fluotar objective (100×/NA1.3) and a CSU 10a Nipkow disc (Yokogawa, Japan); a CoolSnap HQ CCD-camera (Photometrix, Tuscon, AZ, USA); and an Ar/Kr-laser (Innova 70 C Spectrum, Coherent, Santa Clara, CA, USA)^23^. Automated exposure, image quality control and other procedures were performed using Acquarium software^24^. The exposure time and the dynamic range of the camera in the red, green and blue channels (R-G-B) were adjusted to the same values for all slides to obtain quantitatively comparable images. Forty serial optical sections were captured at 0.3-μm intervals along the z-axis.

### Chromatin condensation quantification by the 2D Fourier transform

In addition to analyses using the intensity profiles from the red, green and blue (R-G-B) channels (R: 53BP1; G: γH2AX; B: TO-PRO3, chromatin) described in the previous paragraph, chromatin condensation was also evaluated by the 2D Fourier transform. From a single 2D section (from a 2D projection) of chromatin (blue channel) observed in the confocal microscope, the 2D Fourier transform in polar coordinates was calculated. Results (correlation coefficients) calculated from confocal microscopy data for DMSO + trehalose, DMSO, trehalose, AFP and untreated were used as criterion for chromatin condensation (sufficiently strong and sharp middle frequency component of the signal). Further details see Suppl. Inf.

### Quantification of DSB induction, changes in higher-order chromatin structure and nuclear envelope integrity

The presence and number of γH2AX/53BP1 foci and other γH2AX signals, as well as co-localization of γH2AX and 53BP1 in the nuclei, was determined visually. Changes in higher-order chromatin structure (condensation) after a freeze/thaw cycle were quantified using the intensity profiles from the red, green and blue (R-G-B) channels (R: 53BP1; G: γH2AX; B: TO-PRO3, chromatin) using the ‘RGB Profile Plot’ plugin for ImageJ 1.47 v software (Wayne Rasband, NIH USA, http://imagej.nih.gov/ij) in addition to visual inspection of 3D microscopic images of nuclei and their confocal slices in all three planes. Intensity profiles along line segments demarcated over 0.2-m thick confocal slices of nuclei were determined separately for the R-G-B channels. Each line segment was designed to cover a substantial part of the nucleus and to include nuclear areas showing maximum and minimum chromatin staining. Sharp changes in high amplitude chromatin staining intensity along the line segment indicate structurally and functionally distinct chromatin domains that are well preserved after the freeze/thaw procedure. By contrast, the absence of these changes in chromatin intensity along the line segment (i.e., only slight and slow changes) indicates extensive erosion in the higher-order chromatin structure. Intensive G-peaks (γH2AX) that co-localized with R-peaks (53BP1) mark DSBs. A continuous γH2AX signal that is more constant in intensity and that is not accompanied by a 53BP1 signal indicates apoptotic/necrotic DNA damage. Evaluation of the shape of cell nuclei (maximum area, roundness factor) was determined using the ‘selection’ and ‘analysis’ tools in Adobe Photoshop CS6. Narrowing or disruptions in the nuclear lamina, as visualized using a lamin A/C antibody, plus chromatin (TO-PRO3) leakage out of the nucleus was interpreted as indicating damage to the nuclear envelope.

### Formation of medium with different osmolarities and cells treatments

All procedures have been described in^19^. The osmolarity of standard culture medium is 290 mOsm. To induce reversible artificial chromatin condensation, the cells were incubated for 12 min in hyperosmotic (570 mOsm) medium. Hyperosmotic (hypertonic) medium was prepared by addition of 1 ml 20 × PBS (2.8 M NaCl, 54 mM KCl, 130 mM Na_2_PO_4_, 30 mM KH_2_PO_4_, pH 7.4) to 19 ml DMEM containing 10% FCS. Hypocondensed chromatin in cells was obtained by cell treatment (12 min) in hypoosmotic (hypotonic) medium. Hypoosmotic medium of about 140 mOsm was obtained by diluting standard DMEM medium with an equal quantity of sterile ddH2O. Since chromatin condensation and decondensation started within seconds, washing in physiological salt solution before cell fixation (immuno-fluorescence microscopy) was strictly avoided. For flow cytometry analyses of cell viability, a post-treatment (hypertonic or hypotonic) cultivation of cells in normal (isotonic) medium (30 min or 24 h) was added to allow cell recovery and/or apoptosis induction. Up to about 15 min the hyperosmotic/hypoosmotic treatment has no/minor effect on cell viability and all changes in chromatin structure and cellular processes were reversible.

### Cells preparation for DART-Orbitrap and LC-MS/MS analyses and DESI

Twenty-four hours prior to the measurements, the cells in culture flasks were provided with fresh DMEM medium (37 °C, pH 7.1) supplemented with 100 mM or 200 mM trehalose (Sigma). Consequently, the cells were thoroughly washed (4 × 1 min) in fresh DMEM without trehalose (as proved by LC-MS/MS), harvested by scratching, resuspended in 1 mL of DMEM (1xPBS for DESI), disintegrated by shaking with glass bead tubes 0.5 mm (MO BIO Laboratories, Carlsbad, CA), with microtube homogenizer (BeadBug microtube homogenizer D 1030-E, Benchmark Scientific, Edison, NJ), 3 min, 4000 rpm, all extracts were centrifuged (6000 rpm/15 min, HERMLE Z326K, Germany) to remove cells debris and glass pellets, and 100× diluted for the measurements. Filtered (Nylon filter Mini-UniPrep 0.45 μm pore size, Whatman, UK) cell supernatants were used for DART and LC-MS/MS. For DESI-MS the cells were cultivated the same way as for DART. Cell culture was rinsed and centrifuged. The pellet was loaded onto a nylon membrane Nylon 66, 0.2 μm (Supelco, Bellefonte, PA). 2 μL of cell suspense were loaded using the MICROMAN pipette (Gilson, Villiers-le-Bel, France). Nylon membrane was fixed to the glass slides (Prosolia, Indianapolis, IN) by the means of double-site tape.

### DART-Orbitrap analysis

DART-Standardized Voltage and Pressure Adjustable (SVPA) ion source with tweezer holder module (IonSense, Saugus, MA) was coupled to Orbitrap Elite mass spectrometer (Thermo Fischer Scientific, Bremen, Germany) through the interface evacuated by the diaphragm pump. The DART ion source was operated in the negative ion mode with helium ionizing gas at the pressure 0.55 MPa. The beam was heated to 400 °C, the grid electrode voltage was set to 350 V. The parameters of the mass spectrometer were tuned as follows: capillary voltage 50 V, tube lens voltage 100 V, skimmer voltage 18 V and capillary temperature 300 °C. The acquisition rate was set to 2 spectra/s with mass resolving power of 120,000 FWHM. All DART mass spectra were acquired over a mass range of *m/z* 50–600. Xcalibur software (Thermo Fischer Scientific, Germany) with DART web-based module was used for the instrument operation, data acquisition and processing.

### LC-MS/MS

An Agilent 1200 Series Rapid Resolution LC System (Agilent Technologies, Waldbronn, Germany) consisted of an on-line degasser, a binary pump, a high performance SL autosampler, a thermostated column compartment, a photodiode array UV-vis detector. The system was coupled on-line to an MS detector Agilent Technologies 6460 Triple Quadrupole LC/MS with Agilent Jet Stream. MassHunter (Agilent Technologies, Germany) software was used for the instrument operation, data acquisition and processing. The Unison UK-Amino 250 mm × 3.0 mm, 3μm particle size (Imtakt, Portland, OR) for trehalose separation under isocratic elution was used. The mobile phase consisted of 70% (acetonitrile) and 30% (0.1% acetic acid (v/v)). The flow rate was 0.7 mL/min and column temperature was 60 °C. The column effluent was directly introduced into triple quadrupole mass detector operated in a negative ESI mode. Samples were analyzed by fast chromatography-MS/MS in the multiple reaction monitoring (MRM) mode to maximize sensitivity. Characteristic transition for trehalose was *m/z* 341 → 89.

### DESI-MS imaging experiments

DESI imaging analysis was performed using a Orbitrap Elite (Thermo Fischer Scientific, Bremen, Germany) with a DESI-2D ion source (Prosolia, Indianapolis, IN). Imaging experiments were performed by continuously scanning the surface in the x-direction and stepping in the y-direction (moving opposite to the direction of the spray) at the end of each line. The DESI ion source geometry was as follows: sprayer-to-sample surface distance of 0.5–1.0 mm, sprayer-to-MS inlet distance of 1.0–1.5 mm, spray impact angle of 58° was used, and collection angle of 10°. Solvent MeOH was sprayed at flow rate 3 μL/min in conventional DESI imaging. Typically, a cells slice (10 × 6 mm) was imaged using 55 lines and step size of 100 μm in the y-direction. The lines were scanned at a constant velocity of 72 μm/s. Full scan mass spectra were acquired in negative ion mode over the range of *m/z* 50–450. The total time to record this image was 63 min. BioMap software was used to process the mass spectral data to generate two-dimensional ion images. The optimized MS instrumental parameters used were 6 bar nebulizer (N_2_) pressure, 380 °C heated capillary temperature, 4 kV spray voltage, 60 V tube lens voltage. The ion injection time was 800 ms, and one microscans were averaged for each pixel in the images. DESI-MSI analysis was performed in negative ion mode for trehalose (*m/z* 341.108).

### Statistical analyses

The results were analysed statistically using the Kruskal-Wallis One Way Analysis of Variance on Ranks with p = < 0.05 considered as statistically significant. Positive cases were re-evaluated by the Mann−Whitney Rank Sum test. Number of samples depended on the analysis (about 50 to 2000 cells/sample in 2 or 3 replicas), as indicated.

## Electronic supplementary material

Supplementary Information

Raw Data

## Data Availability

The authors declare that all data supporting the findings of this study are available within the paper and its Supplementary Information files. In addition, all relevant data are available from the authors per request.
