# Supplementary material for: Chromatin architecture changes and DNA replication fork collapse are critical features in cryopreserved cells that are differentially controlled by cryoprotectants
Source: Sci Rep. 2018 Oct 2;8:14694. doi: 10.1038/s41598-018-32939-5 (PMC6168476; doi:10.1038/s41598-018-32939-5)
Supplement: Supplementary file 1 — Supplementary Information [file 41598_2018_32939_MOESM1_ESM.docx]

**Chromatin architecture changes and DNA replication fork collapse are critical features in cryopreserved cells that are differentially controlled by cryoprotectants**

**SUPPLEMENTARY INFORMATION**

Martin Falk^1*^, Iva Falková^1^, Olga Kopečná^1^, Alena Bačíková^1^, Eva Pagáčová^1^, Daniel Šimek^2^, Martin Golan^2,3^, Stanislav Kozubek^1^, Michaela Pekarová^1^, Shelby E. Follett^4^, Bořivoj Klejdus^5,6^, K. Wade Elliott^7^, Krisztina Varga^7^, Olga Teplá^8,9^ and Irena Kratochvílová^2*^

^1^The Czech Academy of Sciences, Institute of Biophysics, Královopolská 135, CZ-612 65 Brno, Czech Republic

^2^The Czech Academy of Sciences, Institute of Physics, Na Slovance 2, CZ-182 21, Prague 8, Czech Republic

^3^Faculty of Mathematics and Physics, Charles University in Prague, Ke Karlovu 5, CZ-121 16 Prague 2, Czech Republic

^4^Department of Chemistry, University of Wyoming, 1000 E. University Ave, Laramie, WY 82071, USA

^5^Institute of Chemistry and Biochemistry, Faculty of Agronomy, Mendel University in Brno, Zemědělská 1, CZ-613 00

^6^CEITEC-Central European Institute of Technology, Mendel University in Brno, Zemědělská 1, CZ-613 00 Brno, Czech Republic

^7^Department of Molecular, Cellular, and Biomedical Sciences, University of New Hampshire, 46 College Road, Durham, NH 03824, USA

^8^ISCARE IVF a.s. Jankovcova 1692, CZ-160 00 Praha 6

^9^VFN Gynekologicko-porodnická klinika, Apolinářská 18, CZ-120 00, Czech Republic

*Corresponding authors: [falk@ibp.cz](mailto:falk@ibp.cz); [krat@fzu.cz](mailto:krat@fzu.cz)

**Table S1 ǀ Proportions [%] of NHDF and MCF7 cells with >30 γH2AX/53BP1 foci/nucleus (confocal microscopy) compared to proportions of S-phase cells (flow cytometry), γH2AX-positive cells (flow cytometry) and Annexin V/PI-positive cells (flow cytometry ** γH2AX-positive and S-phase cells were quantified by flow cytometry (FC) using γH2AX/H2AX and Propidium iodide (PI, DNA content) staining, respectively. Experiments were performed 30 min after cryoprotectant treatment and/or freezing/thawing (F/T). Immunofluorescence confocal microscopy (CM). The values are the means with standard errors.

| treatment | | Cells (30 min post-treatment and/or freezing/thawing) | | | |
| --- | --- | --- | --- | --- | --- |
|  |  | with >30 γH2AX/53BP1 foci (CM) | in S-phase (PI staining; FC) | γH2AX- positive (FC) | annexin V/PI positive  (FC) |
| **NHDF cells** | | | | | |
| non frozen | untreated | <0.5 | 8.2 ± 2.5 | 4.8 ± 1.1 | 18.0 ± 1.0 |
|  | T | <0.5 | 5.5 ± 1.5 | 9.8 ± 0.4 | 26.3 ± 2.9 |
|  | DMSO | <0.5 | 6.9 ± 1.7 | 4.3 ± 0.8 | 30.3 ± 1.5 |
|  | DMSO+T | <0.5 | 5.3 ± 2.2 | 4.3 ± 0.4 | 28.4 ± 2.9 |
| post F/T | untreated | 9.0 ± 0.5 | 7.7 ± 2.1 | 37.0 ± 7.7 | 86.1 ± 4.4 |
|  | T | 3.8 ± 0.2 | 4.4 ± 0.6 | 31.3 ± 3.0 | 51.9 ± 5.0 |
|  | DMSO | 5.2 ± 0.4 | 8.3 ± 1.8 | 10.3 ± 1.8 | 31.9 ± 2.2 |
|  | DMSO+T | 4.0 ± 0.2 | 5.3 ± 1.5 | 6.3 ± 0.7 | 30.8 ± 2.5 |
| **MCF7 cells** | | | | | |
| non frozen | untreated | <0.5 | 13.4 ± 0.2 | 7.1 ± 0.7 | 17.5 ± 1.4 |
|  | T | <0.5 | 8.0 ± 0.5 | 9.6 ± 2.8 | 38.4 ± 5.6 |
|  | DMSO | <0.5 | 14.7 ± 1.3 | 8.7 ± 3.6 | 18.9 ± 1.1 |
|  | DMSO+T | <0.5 | 7.3 ± 0.5 | 10.7 ± 5.1 | 38.2 ± 7.2 |
| post F/T | untreated | 13.8 ± 2.1 | 14.3 ± 0.4 | 54.5 ± 14.8 | 99.1 ± 0.2 |
|  | T | 6.2 ± 2.2 | 9.5 ± 0.2 | 45.3 ± 10.1 | 85.7 ± 1.0 |
|  | DMSO | 16.0 ± 2.0 | 15.8 ± 0.3 | 20.9 ± 9.9 | 23.7 ± 1.0 |
|  | DMSO+T | 7.3 ± 1.3 | 10.2 ± 0.6 | 15.3 ± 2.5 | 23.9 ± 1.5 |

**Table S2 ǀ** **Correlation between condensed chromatin, determined by confocal fluorescence microscopy (TO-PRO-3 staining) in combination with image 2D Fourier transform**, and cell survival quantified by flow cytometry (Annexin V + PI positivity) before freezing and at 30 min and 24 h after thawing. Upper table – NHDF cells; bottom table – MCF7 cells.

Note: In our previous work^3^ (PMC5602551), only the data on the viability of fibroblasts 24 h post treatment were studied. In the present study, we have evaluated cell viability for NHDF fibroblasts and MCF7 cells at two time points post freezing/thawing (30 min and 24 h); this is important to obtain more detailed image about the freezing/thawing effects. In the present study, all the viability values were obtained with new software (Guava InCyte soft. 3.1.1., Millipore) allowing us more precise analyses. First, we used the same gates in all experiments, and second, in all cases, we excluded cell debris more precisely (as compared to RSC Adv.)^3^. The same gating and the same style of debris exclusion is extremely important in viability evaluation of frozen/thawed cells because many thawed cells are more or less fragmented. With the software used in RSC Adv. (MUSE machine original software) this was not feasible.

|  | Condensed chromatin [% of cells] | | Viable cells [%] (flow-cytometry) | |
| --- | --- | --- | --- | --- |
| treatment: | before freezing/thawing | 30 min after freezing/thawing | 30 min after freezing/thawing | 24 h after freezing/thawing |
| **NHDF cells** | | | | |
| untreated | 16.7 ± 2.9 | 12.0 ± 2.5 | 13.9 ± 4.4 | 22.2 ± 2.1 |
| AFP | 25.8 ± 5.1 | 15.1 ± 4.6 | 31.5 ± 4.8 | 35.4 ± 5.3 |
| trehalose | 21.1 ± 6.4 | 43.5 ± 6.9 | 48.1 ± 5.1 | 60.3 ± 3.5 |
| DMSO | 56.7 ± 9.2 | 73.2 ± 2.5 | 68.1 ± 2.2 | 79.0 ± 7.0 |
| DMSO + trehalose | 52.3 ± 6.4 | 82.5 ± 5.0 | 69.2 ± 2.5 | 65.8 ± 2.2 |
| **MCF7 cells** | | | | |
| untreated | 8.6 ± 2.8 | 7.9 ± 2.3 | 0.9 ± 0.2 | 0.6 ± 0.8 |
| trehalose | 12.0 ± 2.2 | 16.0 ± 2.8 | 14.3 ± 0.5 | 9.2 ± 1.0 |
| DMSO | 31.6 ± 5.1 | 59.7 ± 2.8 | 76.3 ± 1.0 | 80.0 ± 0.5 |
| DMSO + trehalose | 39.5 ± 4.7 | 62.0 ± 5.8 | 76.2 ± 1.5 | 66.5 ± 1.0 |

**
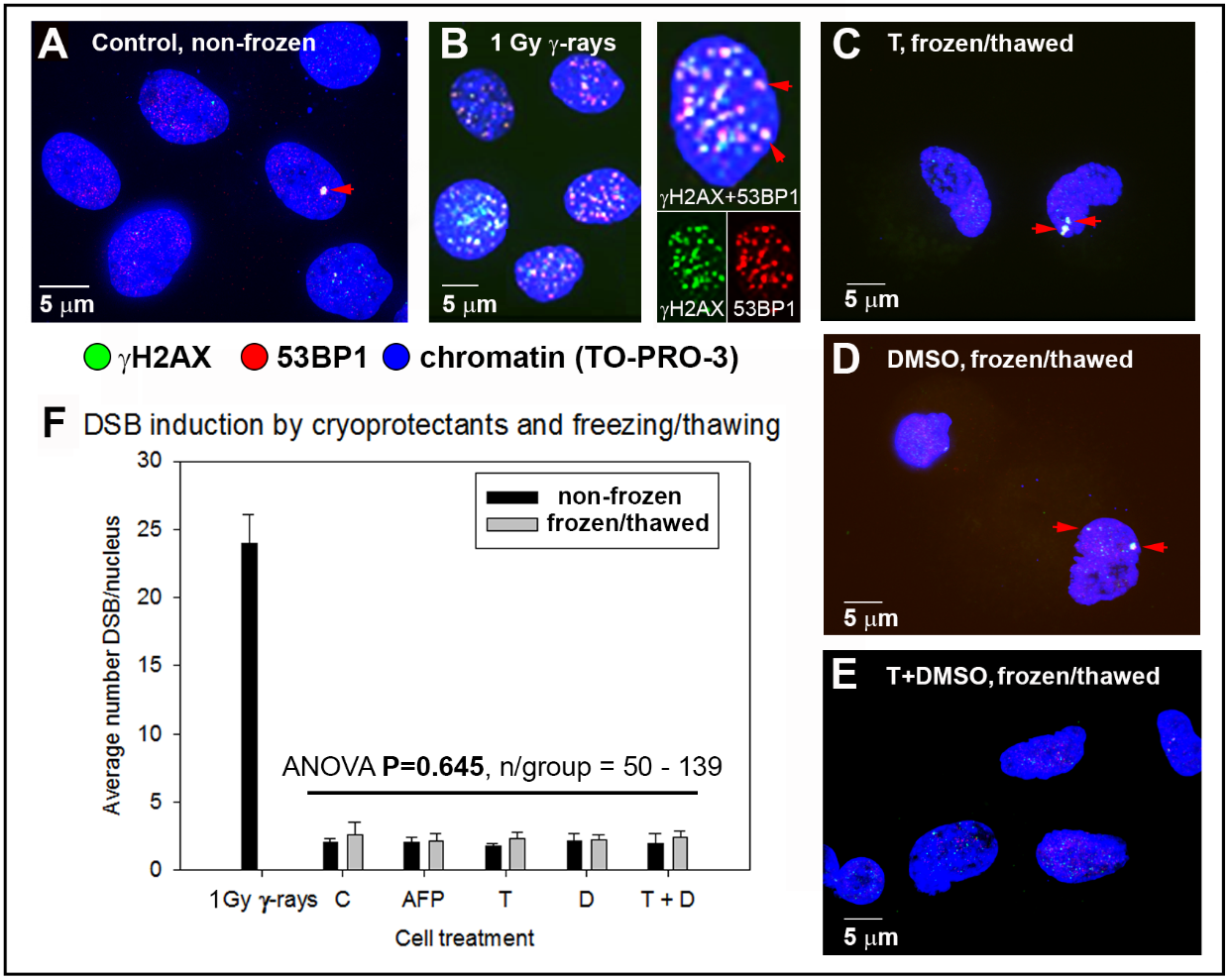
**

**Fig. S1 ǀ DSB induction in a sub-fraction of frozen/thaw NHDF cells with <30 DSB that were treated or not treated with the indicated cryoprotectants.** Images show 40 superimposed 0.2-μm thick confocal slices. DSBs are marked by γH2AX (green) colocalizing with 53BP1 (red). **A.** positive control cells were irradiated with 1 Gy of γ-rays and were not frozen; the right panel shows a detail of γH2AX foci and 53BP1 foci co-localization, marking the DSB lesions. **B.** Negative controls were not frozen; **C-D.** Cells were frozen/thawed in the presence of the cryoprotectants (**C**, trehalose; **D,** DMSO; **E,** trehalose+DMSO). Chromatin is counterstained with TO-PRO3 (blue). **F.** Average DSB foci numbers per nucleus determined 30 min PT for cells treated or not treated with cryoprotectants and submitted to freezing/thawing. Average numbers of DSB foci and their standard deviations were calculated only for nuclei with <30 foci/nucleus in order to compare DSB induction in predominate fractions of cells before and after freezing/thawing. In unfrozen and frozen/thawed states, these fractions corresponded to about 99 % and 91 % of the cell population, respectively. The *P* value was obtained using the Kruskal-Wallis One Way Analysis of Variance on Ranks for all samples except for the γ-ray irradiated sample (n = 50–139 nuclei).


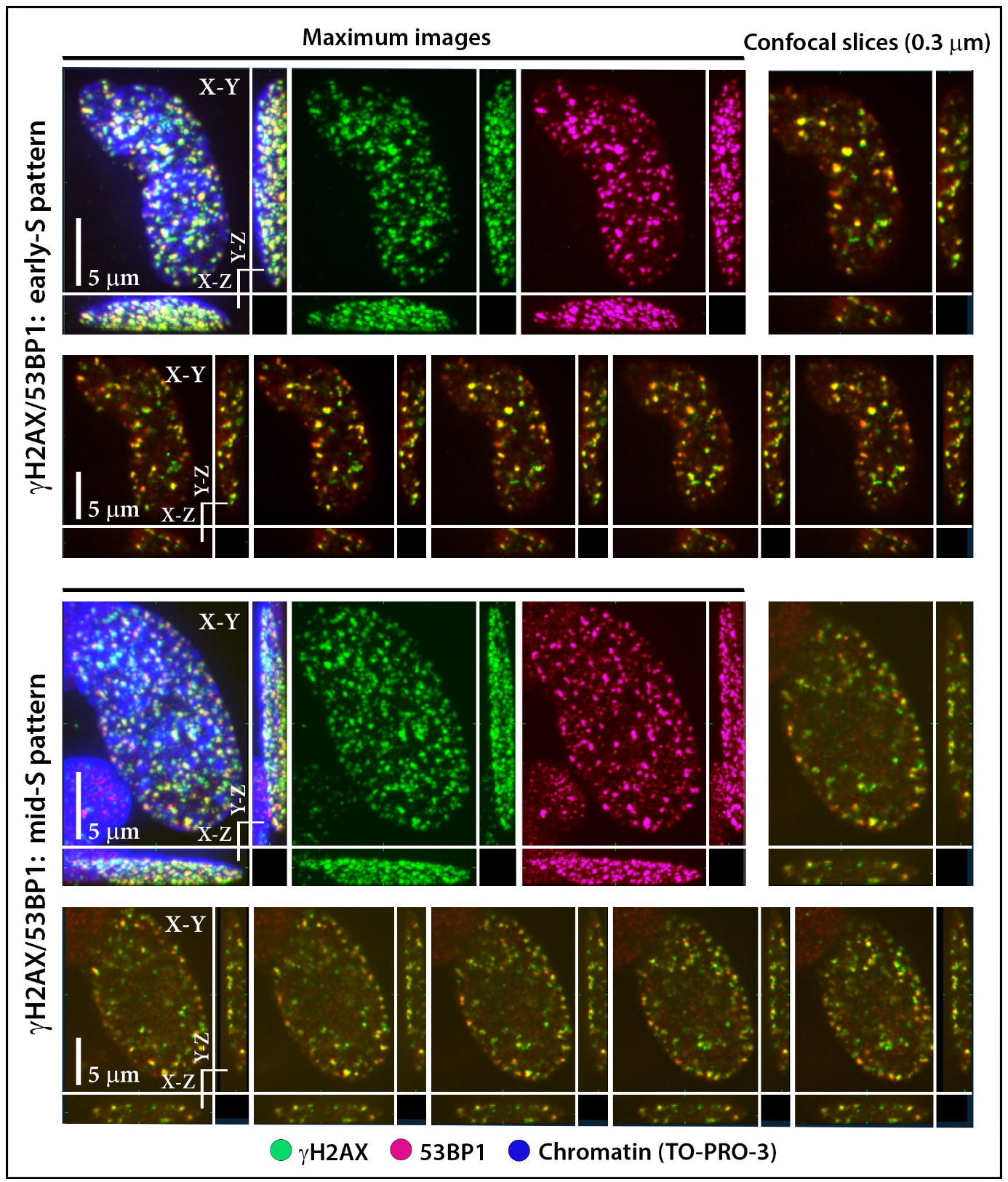


**Fig. S2 ǀ Immunofluorescence confocal images of NHDF cells with >30 γH2AX/53BP1 foci/nucleus that emerged after freezing/thawing.** Top panel: A nucleus with >100 small DSB foci dispersed throughout the nucleus in a pattern resembling that of replication sites in the early S-phase. Lower panel: A nucleus, again with >100 small DSB foci, but distributed especially along the nuclear rim (i.e. in a pattern resembling that of replication sites in the mid S-phase). Upper lines of images: 3D projections (x-y, x-z and y-z) of maximum images composed of 40 confocal slices, each 0.3 μm-thick (left column), plus an enlarged confocal slice plus (the rightmost images). Lower lines of images: five consecutive single confocal slices, 0.3 μm-thick (remaining columns). γH2AX – green, 53BP1 – purple, blue – chromatin staining (TO-PRO3).

**
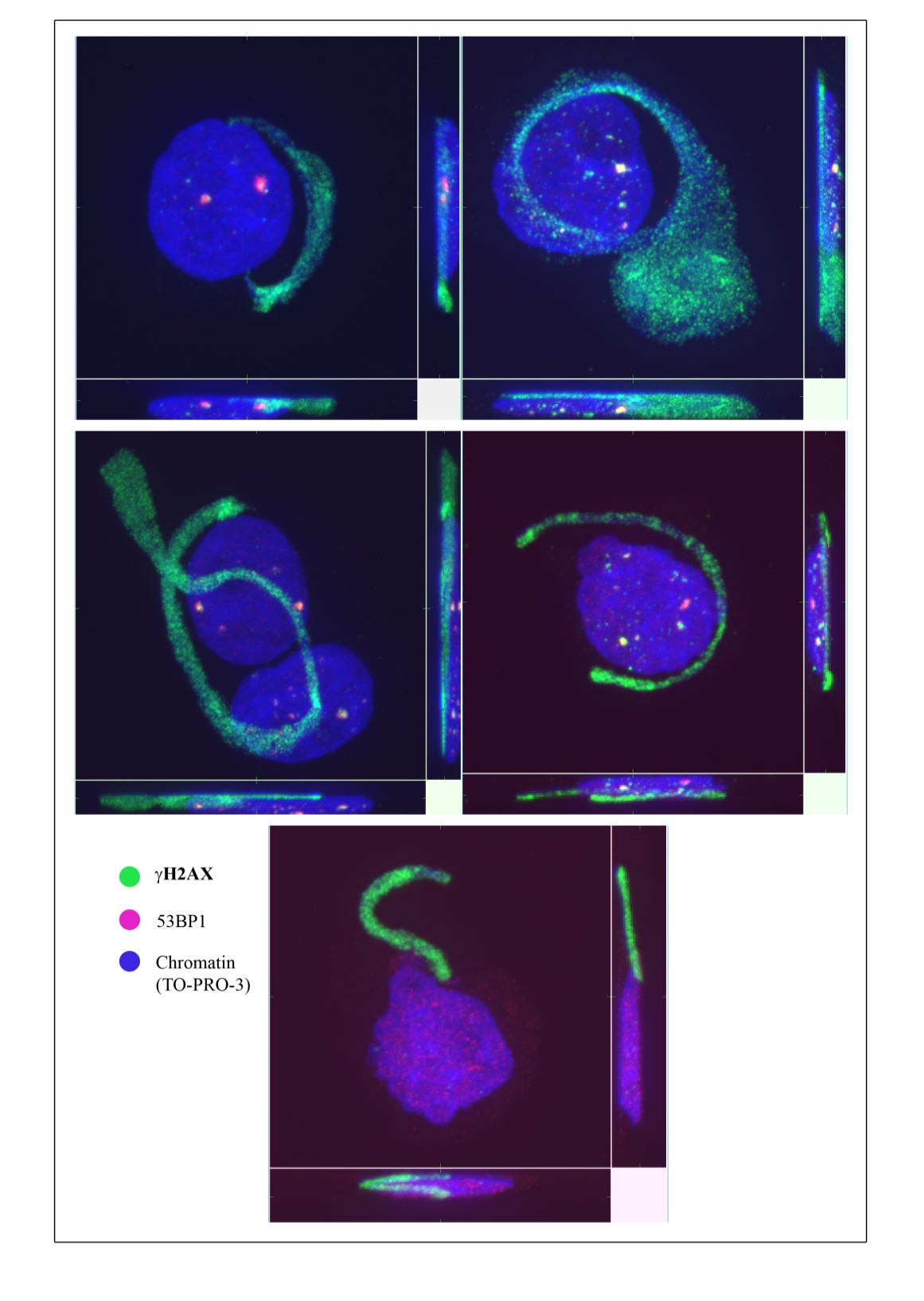
**

**Fig. S3 ǀ Chromatin perturbations (out of interrupted nuclei) caused by freeze/thaw processes and marked by γH2AX.** Visible is also altered (decondensed) higher-order chromatin structure (blue, TO-PRO3) while DSBs (colocalized γH2AX and 53BP1 signal, green + red) are detected in the extent corresponding to never frozen cells. Images consist of 40 superimposed 0.2-μm thick confocal slices; magnification 100×).


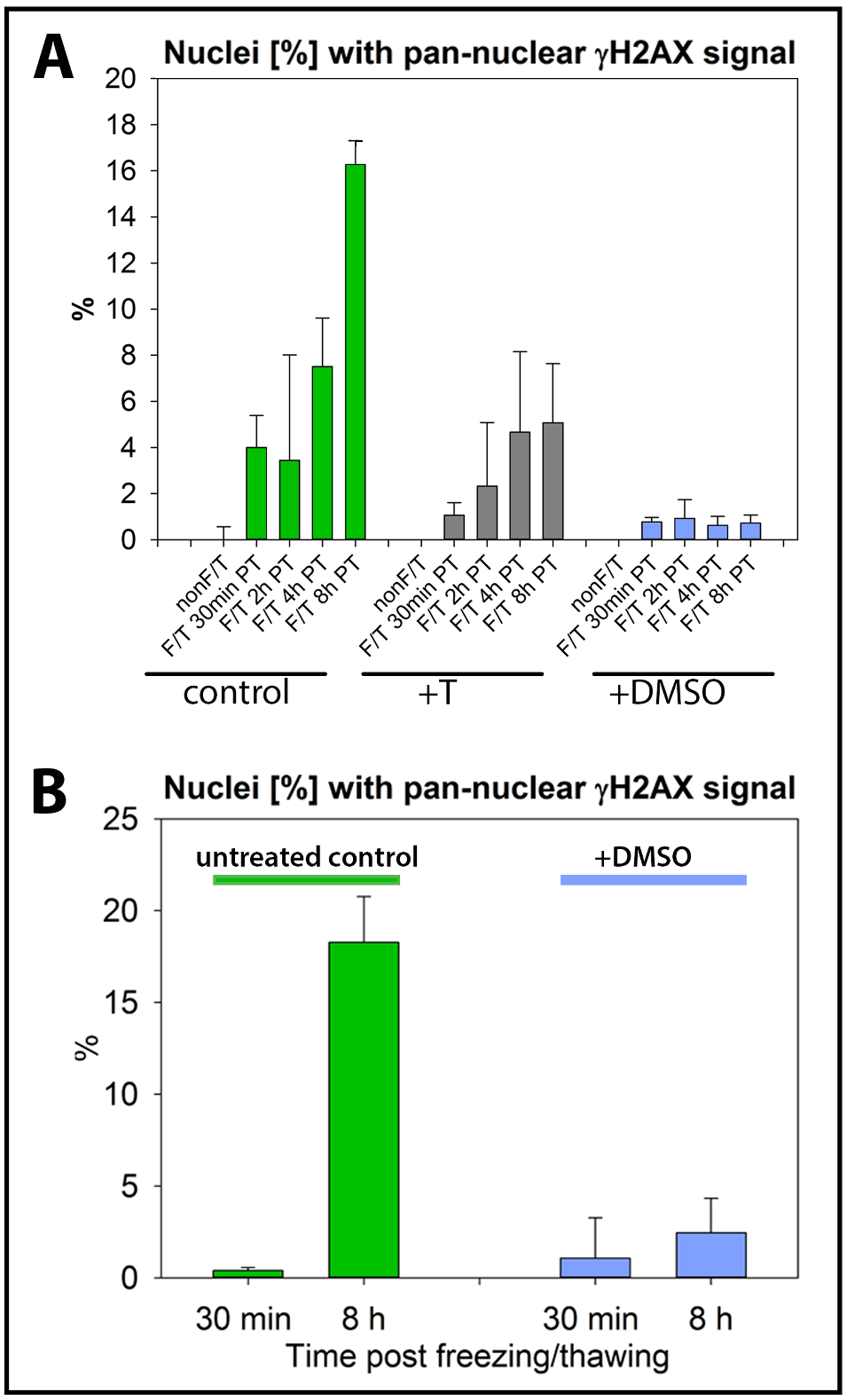


**Fig. S4** **ǀ Proportion [%] of MCF (A) NHDF (B) cells with pan-nuclear γH2AX staining prior to freezing/thawing and at the indicated periods of time after thawing for cells incubated or not incubated with the cryoprotectants.**


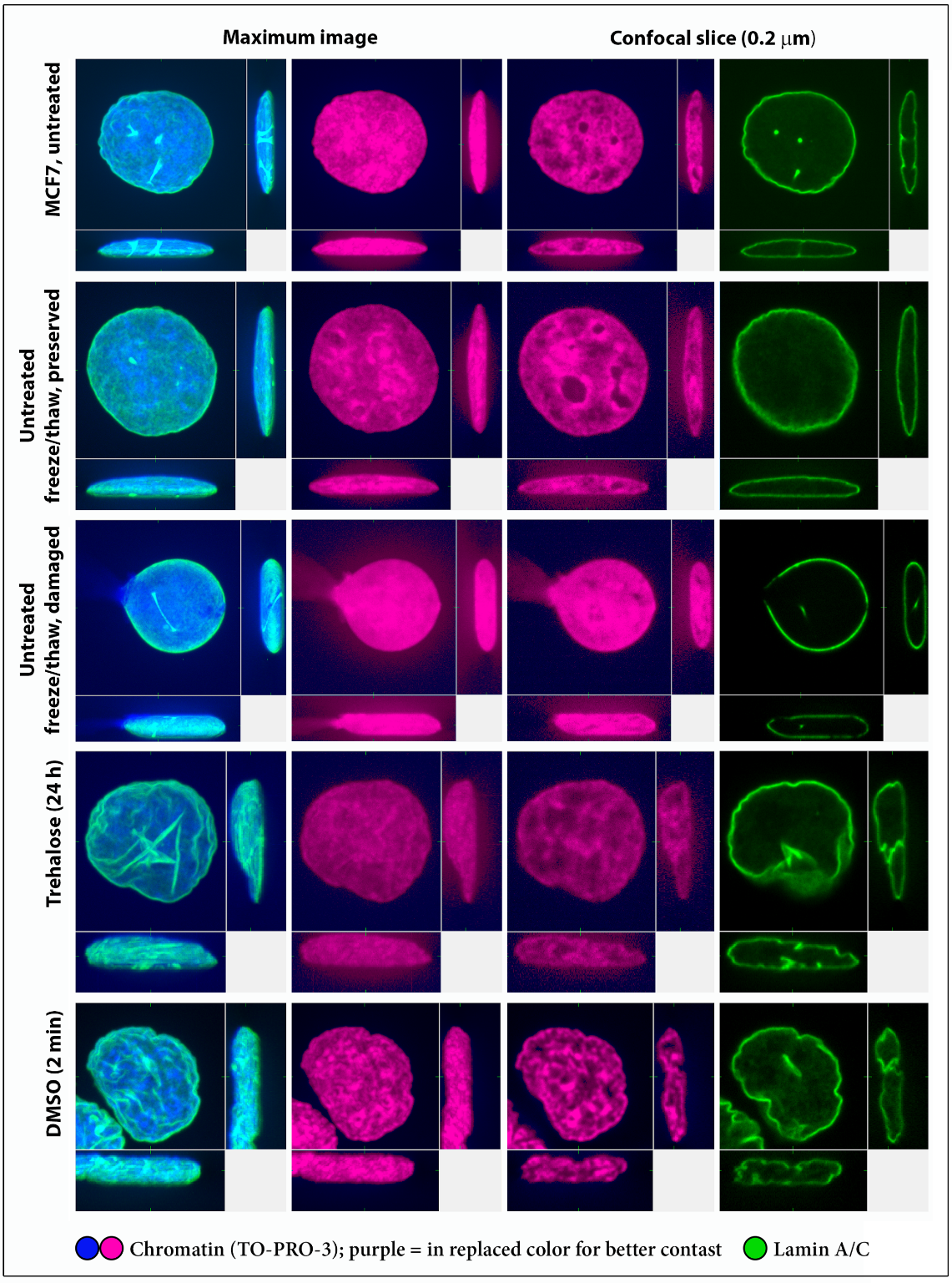


**Fig. S5 ǀ The effects of a freeze/thaw cycle on the higher-order chromatin structure and nuclear envelope in mammary carcinoma MCF7 cells in the presence of cryoprotectants.** Top row: untreated control cells that were not frozen. Other rows: cells frozen in the presence of the indicated cryoprotectant. ‘Maximum images’ are composed of 40 superimposed 0.2-μm thick confocal slices and shown together with x-z and y-z projections (left columns). Left columns present single confocal slices (0.2-μm thick) through the cell nucleus (performed at the central nuclear plane) with their x-z and y-z projections. Nuclear envelopes were visualized using lamin A/C antibody (green), and the chromatin was counterstained with TO-PRO3 (blue or violet to better visualize the chromatin structure).

**
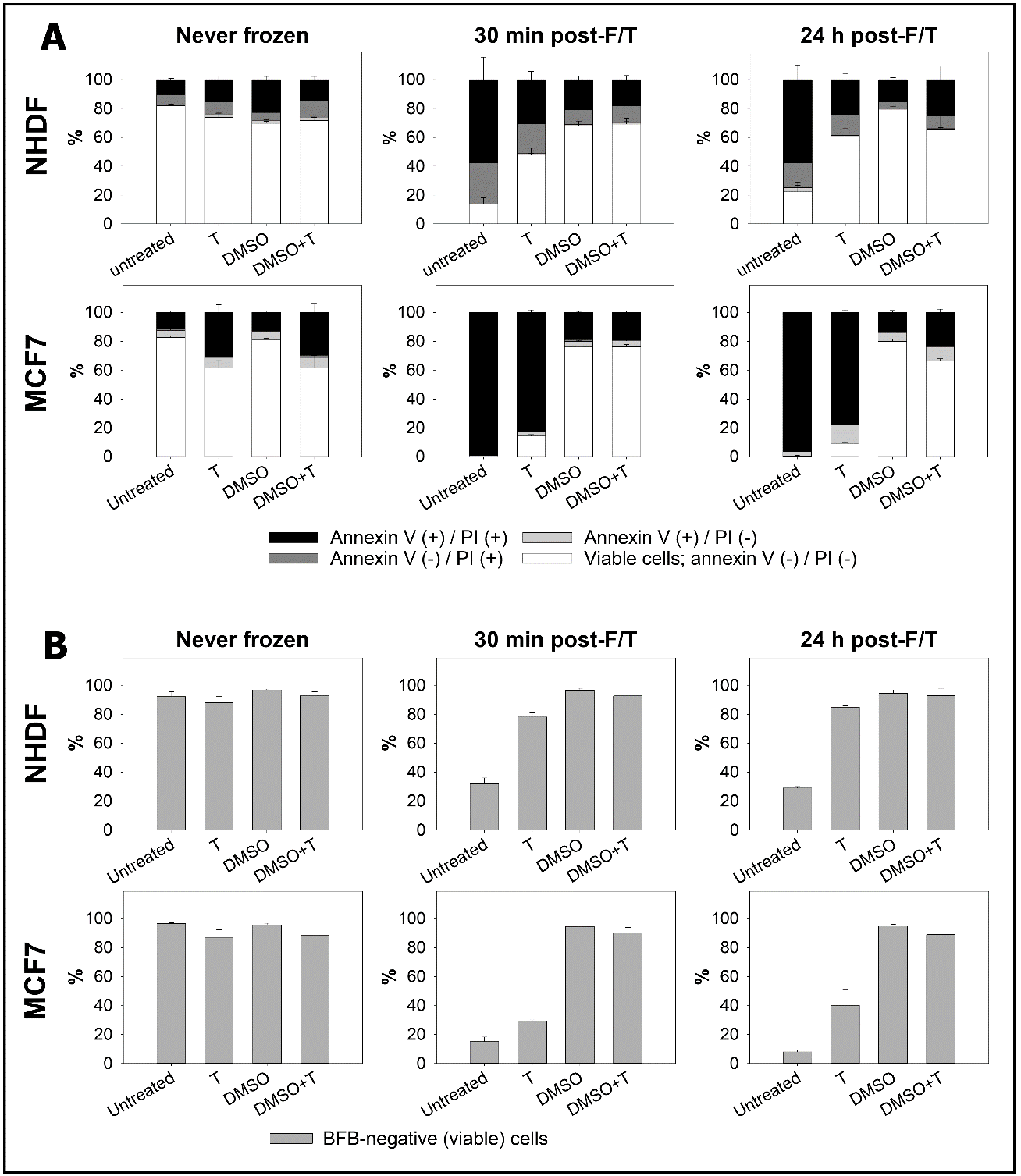
**

**Fig. S6 ǀ** **Detailed analyses of NHDF cells’ dying upon freezing/thawing in presence or absence of trehalose, DMSO or combination of both cryoprotectants.** **A**. Flow cytometry with Annexin V + Propidium iodide (PI), 24 h post-thawing. B. Viability of untreated and DMSO, trehalose, DMSO+trehalose treated never frozen and frozen/thawed MCF7 and NHDF cells. Note: In our previous work^3^ (PMC5602551), only the data on the viability of fibroblasts 24 h post treatment were studied. In the present study, we have evaluated cell viability for NHDF fibroblasts and MCF7 cells at two time points post freezing/thawing (30 min and 24 h); this is important to obtain more detailed image about the freezing/thawing effects. In the present study, all the viability values were obtained with a new software (Guava InCyte soft. 3.1.1., Millipore) allowing us more precise analyses. First, we used the same gates in all experiments, and second, in all cases, we excluded cell debris more precisely (as compared to RSC Adv.)^3^. The same gating and the same style of debris exclusion is extremely important in viability evaluation of frozen/thawed cells because many thawed cells are more or less fragmented. With the software used in RSC Adv. (MUSE machine original software) this was not feasible.


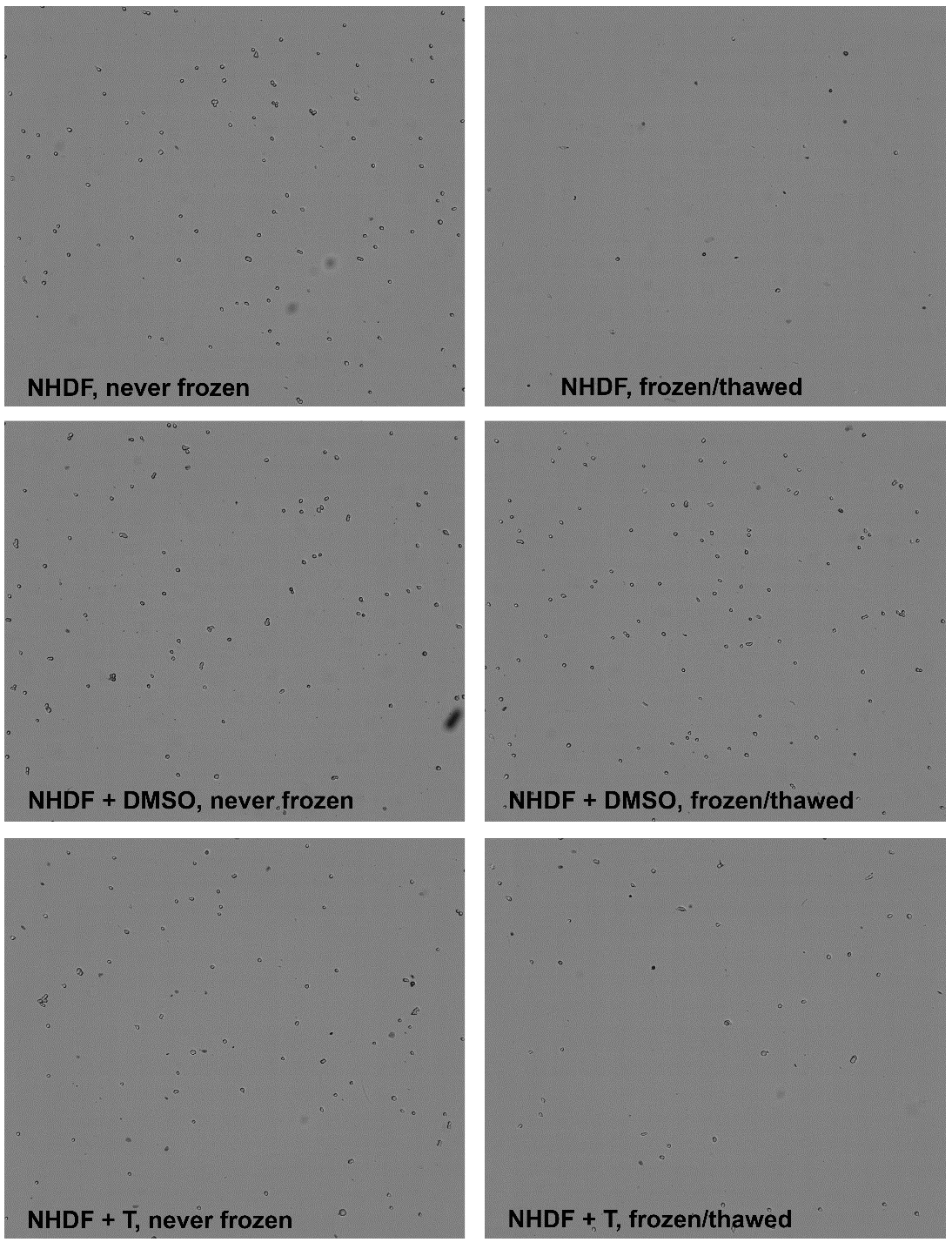


**Fig. S7** **ǀ Detailed analyses of NHDF cells’ dying upon freezing/thawing in presence or absence of trehalose, DMSO or combination of both cryoprotectants.** Flow cytometry (Fig. S6) with criterion (7.5×10^-6^, when unity is the zero frequency maximum), which assured 90% of nuclei in testing samples being properly qualified in accordance with the experienced staff judgment.

**Chromatin condensation quantification**

Fourier transform composes from a three basic components: i) the zero and low spatial frequency component coming from the rotationally averaged size of the whole nucleus, ii) the middle frequency component coming from the condensed chromatin, and iii) the broad frequency background coming mostly from the noise recorded. From a single 2d section (from a 2d projection) of chromatin (blue channel) observed in the confocal microscope, the 2d Fourier transform in polar coordinates was calculated.

,

where *b*(r,α) represents the luminosity of blue channel in polar coordinates. Only the radial part of *c*(ρ,α) was considered, while the phase of the Fourier transform was forgotten and the function was normalized have maximum equal to one:

.

The resulting radial part of F.T. *y*(ρ) then composes from a three basic components: i) the zero and low spatial frequency component coming from the rotationally averaged size of the whole nucleus, ii) the middle frequency component coming from the condensed chromatin, and iii) the broad frequency background coming mostly from the noise recorded. The low frequency peak has always its maximum for zero frequency (ρ=0), while the middle frequency component, if a dominant condensation length *L*_c_ occurs, can show a peak for a frequency inversely proportional to the condensation length ρ_m_=1/ *L*_c_. We fit the whole radial part of the spectrum by these three components represented by 8 parameters:

.

It is the sum of two Pearson VII functions plus c constant background, the interesting middle-frequency component being the second term. The examples of three different nuclei, classified as those with condensed chromatin, normal chromatin and damaged (after freezing-thawing) nucleus are shown in Figure S11. As obvious, the middle frequency component (black dots) is the strongest for nucleus with condensed chromatin.

For discrimination of nuclei with condensed chromatin, the best criterion appears to look for the first momentum of the middle frequency component:

,

Where the *y_MF_* represents either the calculated middle-frequency component from the refinement or the difference of the experimental profile from the other components (i.e. black dots in Fig. S8). The best results gave the product of both these. With all the images of the same magnification, a threshold value of the first momentum gave a good discrimination criterion (7.5×10^-6^, when unity is the zero frequency maximum), which assured 90% of nuclei in testing samples being properly qualified in accordance with the experienced staff judgment.


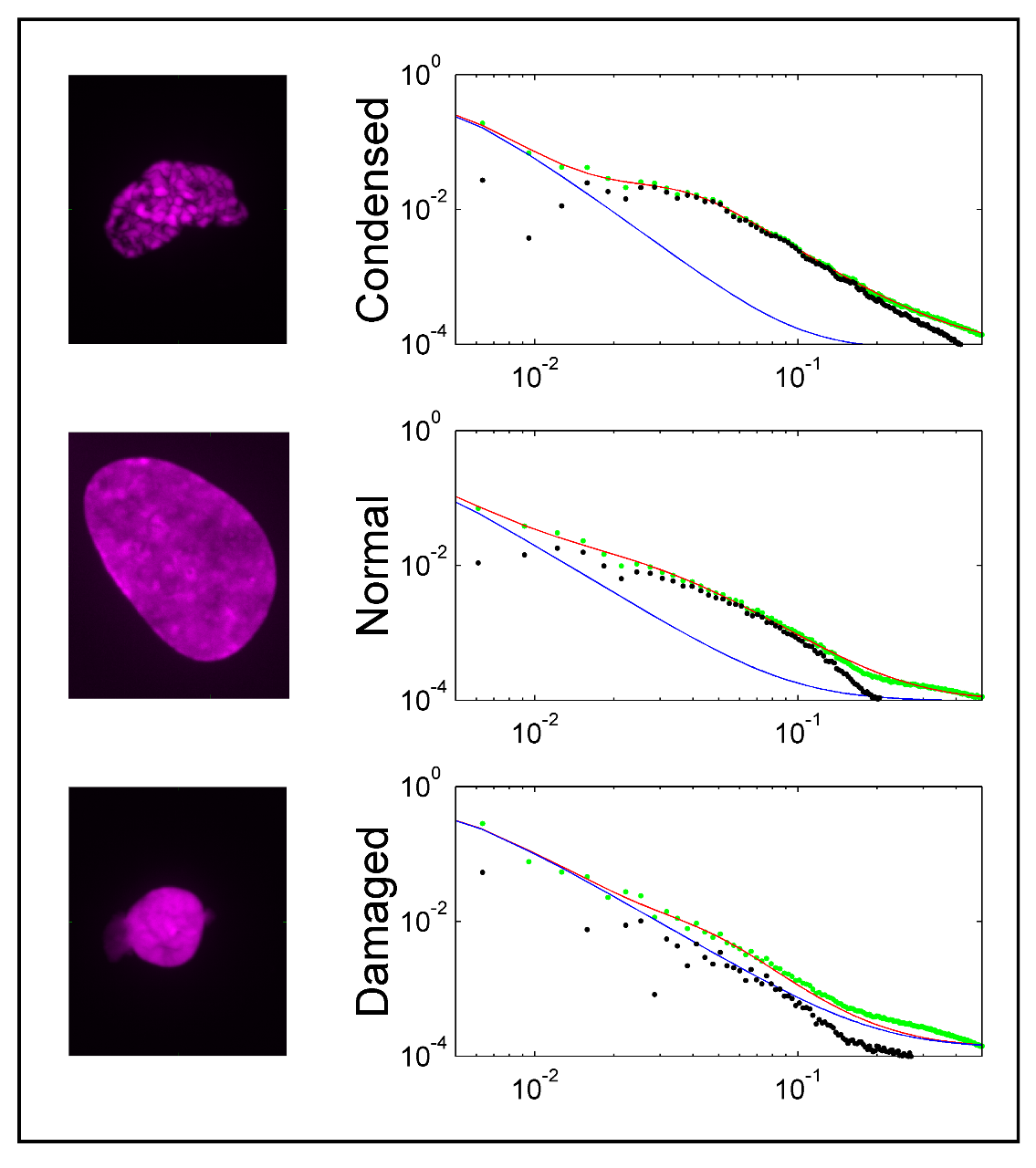


**Fig. S8** **ǀ Examples of nuclei (left) with (top to bottom) condensed, normal, and damaged/structureless chromatin with corresponding radial profile of Fourier transform intensity in log-log scale (right).** Green dots are data calculated from the pictures, blue line represents the low-frequency component and the background, red line includes also a middle frequency component. Black dots show the difference of measured data from the blue line.


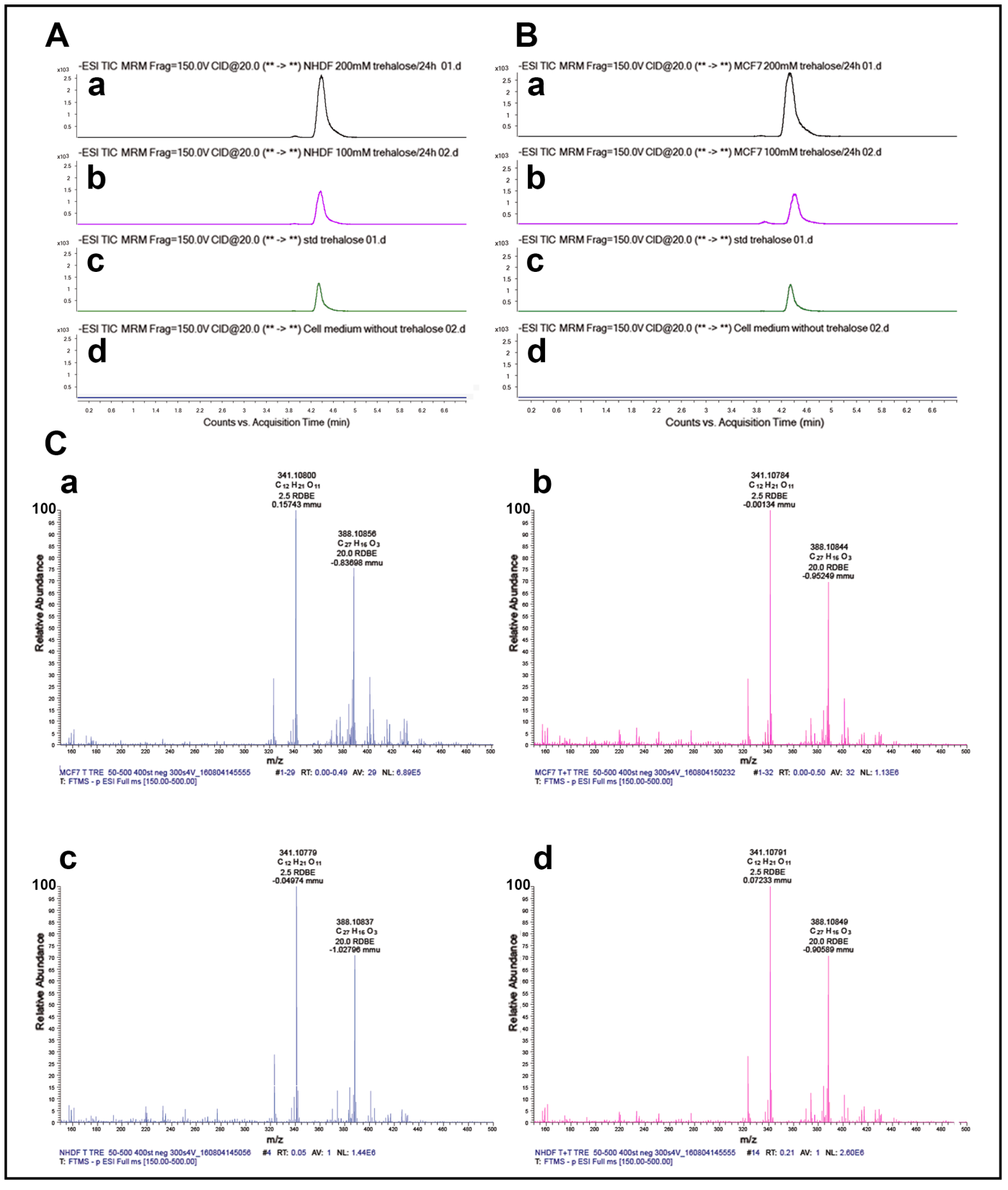


**Fig. S9** **ǀ LC MS/MS + DART-Orbitrap analysis of trehalose in NHDF and MCF7 cells.** **A**+ **B**: Negative ion mode of MRM chromatograms: **a**. NHDF cells with 200 mM trehalose/24 h; **b**. NHDF cells with 100 mM trehalose/24 h; **c**. standard of trehalose; **d**. Cells medium without trehalose **B**. MCF7 cells (a-d as for A). **C**. DART: **a**. MCF7 cells incubated with 100 mM trehalose for 24 h; **b**. MCF7 cells incubated with 200 mM trehalose for 24 h; **c**. NHDF cells incubated with 100 mM trehalose for 24 h; d. NHDF cells incubated with 200 mM trehalose for 24 h.


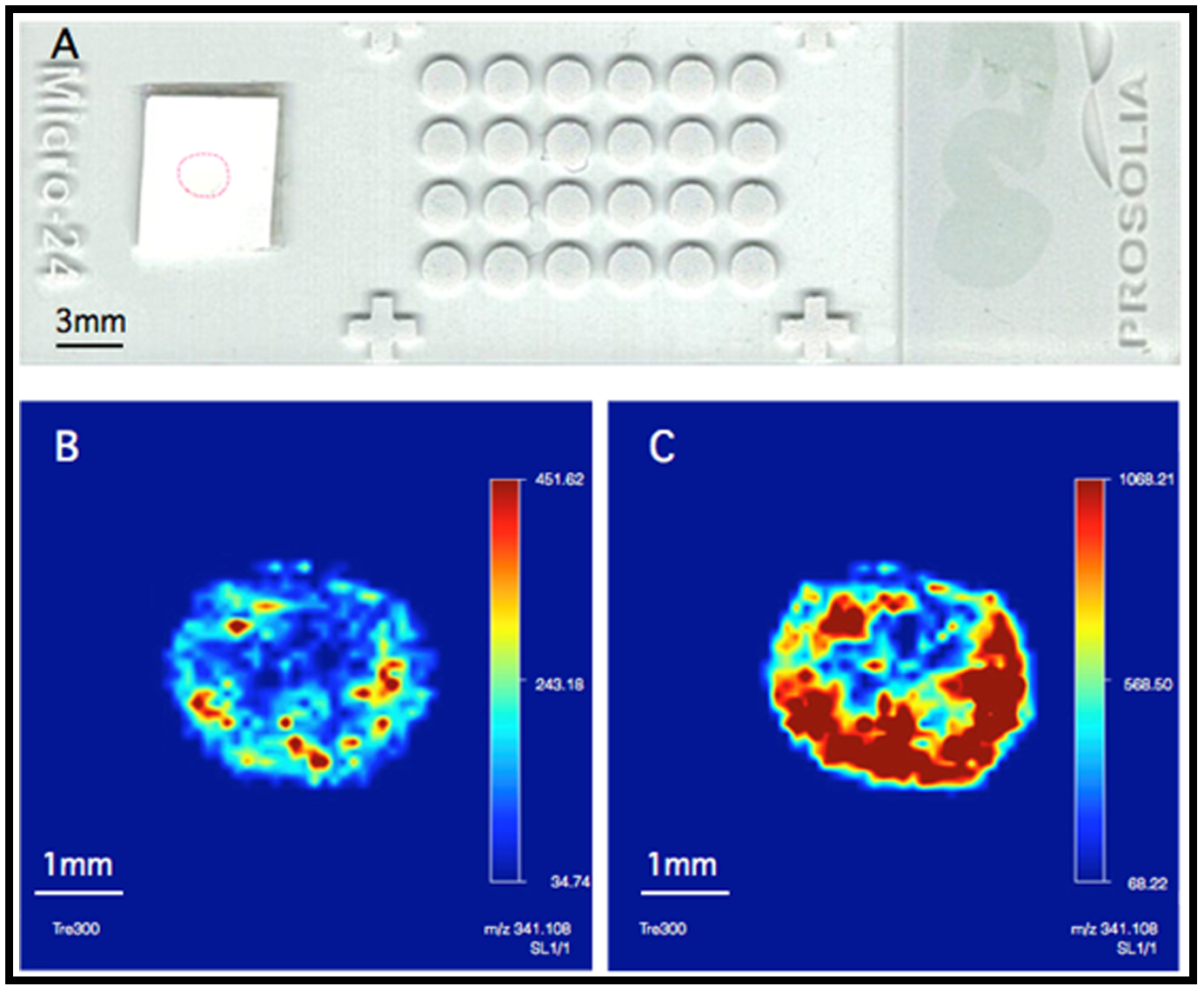


**Fig. S10** **ǀ DESI-MS images showing the cell absorption and relative ion intensity of trehalose (m/z 341.108) by/in NHDF cells.** Displayed are spots of cells incubated for 24 h with **A:** Cell culture loaded onto a nylon membrane, fixed to the glass slide by the means of double-site tape**.** Displayed are spots of cells incubated for 24 h with: **B –** 100 mM trehalose; or **C –** 200 mM trehalose. Trehalose is being absorbed by cells in the time course of our experiments; blue-to-red scale indicates increasing concentrations of trehalose in cells. See Fig. S9 for LC-MS/MS and DART results.


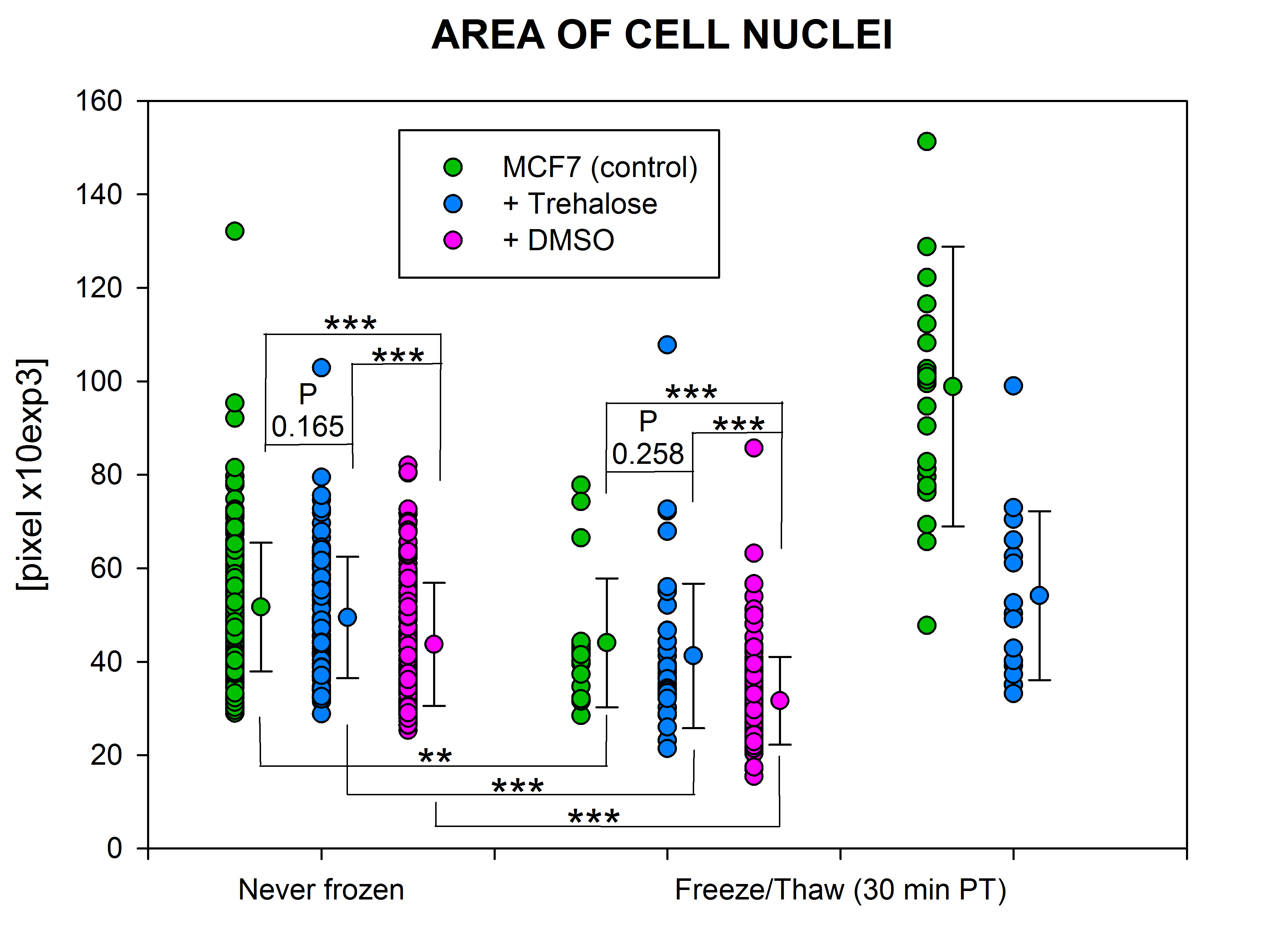


**Fig. S 11 ǀ MCF7 cell nuclei condensation upon cell incubation with cryoprotectants studied, before and after freeze/thaw.** Nuclear areas [pixels .10^3^]) on maximum images are displayed for all cell nuclei together with means ± SE. Two rightmost data distributions are for cells with highly damaged cell nuclei (that are almost absent for DMSO treatment and therefore not displayed) while remaining distributions are for preserved nuclei (*) = P<0.05; (**) = P<0.01; (***) = P<0.001; Mann-Whitney Rank Sum Test.


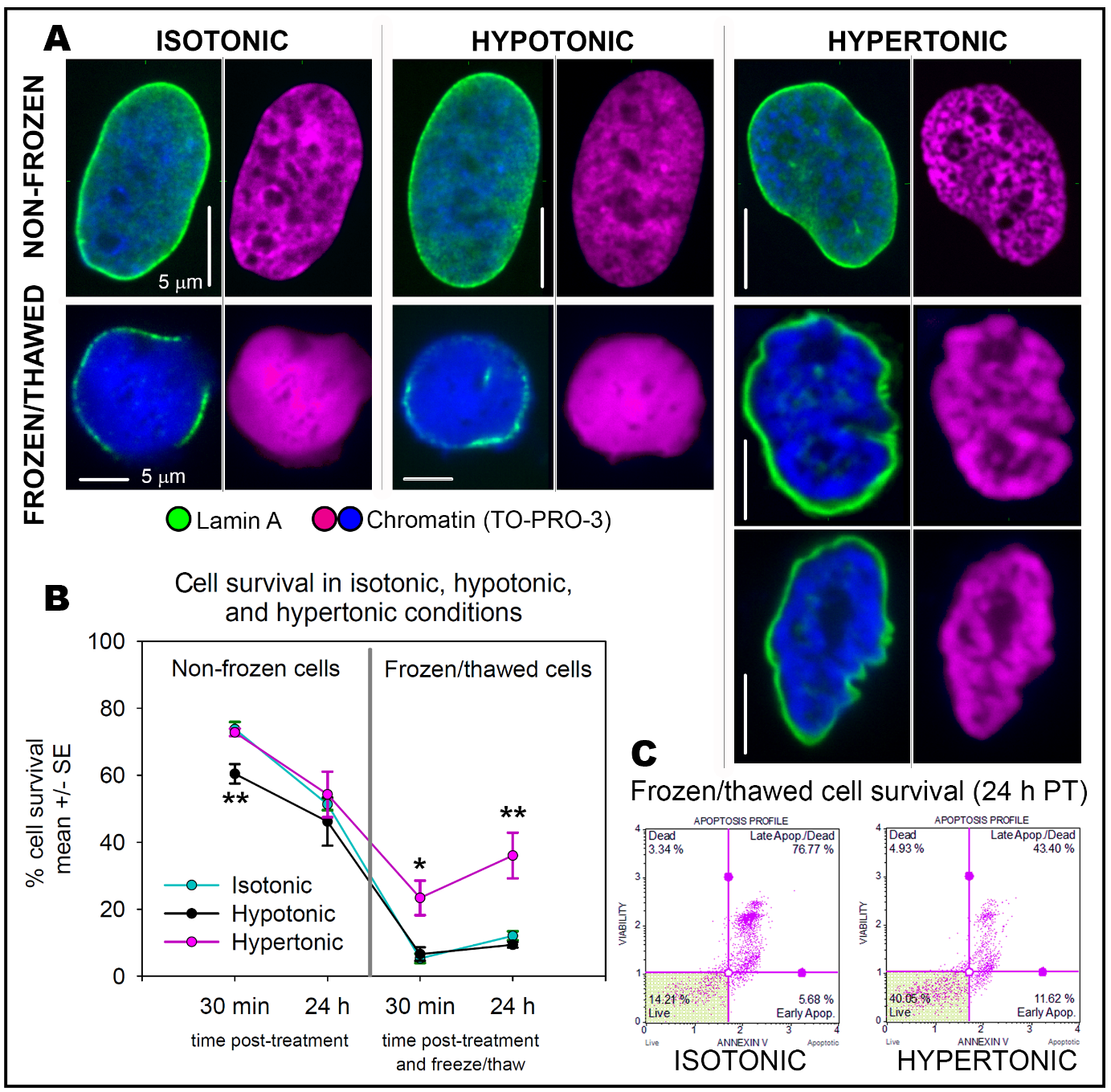
**Fig. S12 ǀ Impact of short pre-freeze incubation of NHDF cells in media with different osmolarities on higher-order chromatin structure, nuclear envelope quality and cell viability.** Before freezing, cells were incubated for 12 min in hypertonic (570 mOsm), hypotonic (140 mOsm), or normal culture medium (isotonic control, 290 mOsm). (A) Higher-order chromatin structure and nuclear envelope quality of non-frozen (top line) and frozen/thawed skin fibroblasts (NHDF), determined by high-resolution confocal immunofluorescence microscopy immediately (without changing the media) after the incubation or after the incubation and freezing/thawing. Nuclear envelopes were visualized using lamin A/C antibodies (green), and the chromatin was counterstained with TO-PRO-3 (blue or purple to better visualize the chromatin structure). (B) Viability of control NHDF (isotonic medium) and NHDF pre-incubated for 12 min in hypotonic or hypertonic medium before and after a freeze/thaw cycle as determined by flow cytometry (see C). After freezing/thawing, cells were transferred to normal (isotonic) medium and cultured for 30 min or 24 h before viability was assessed. Error bars correspond to the SE; statistically significant (unpaired two-tailed t-test) differences relative to control (isotonic) cells are indicated by asterisks (*, P < 0.05; **, P < 0.01; ***, P < 0.001). (C) Representative flow cytograms for NHDF cells that were frozen/thawed in isotonic (left) and hypertonic (right) medium. Horizontal axis: Annexin V staining, vertical axis: 7-AAD staining (bottom-left corner contains viable cells).
