# Supplementary material for: Chromatin architecture changes and DNA replication fork collapse are critical features in cryopreserved cells that are differentially controlled by cryoprotectants
Source: Sci Rep. 2018 Oct 2;8:14694. doi: 10.1038/s41598-018-32939-5 (PMC6168476; doi:10.1038/s41598-018-32939-5)
Supplement: Supplementary file 2 — Raw Data [file 41598_2018_32939_MOESM2_ESM.docx]

**Martin Falk^1*^, Iva Falková^1^, Olga Kopečná^1^, Alena Bačíková^1^, Eva Pagáčová^1^, Daniel Šimek^2^, Martin Golan^2,3^, Stanislav Kozubek^1^, Michaela Pekarová^1^, Shelby E. Follett^4^, Bořivoj Klejdus^5,6^, K. Wade Elliott^7^, Krisztina Varga^7^, Olga Teplá^8,9^ and Irena Kratochvílová^2*^**

^1^ The Czech Academy of Sciences, Institute of Biophysics, Královopolská 135, CZ-612 65 Brno, Czech Republic

^2^ The Czech Academy of Sciences, Institute of Physics, Na Slovance 2, CZ-182 21, Prague 8, Czech Republic

^3^ Faculty of Mathematics and Physics, Charles University in Prague, Ke Karlovu 5, CZ-121 16 Prague 2, Czech Republic

^4^ Department of Chemistry, University of Wyoming, 1000 E. University Ave, Laramie, WY 82071, USA

^5^ Institute of Chemistry and Biochemistry, Faculty of Agronomy, Mendel University in Brno, Zemědělská 1, CZ-613 00

^6^ CEITEC-Central European Institute of Technology, Mendel University in Brno, Zemědělská 1, CZ-613 00 Brno, Czech Republic

^7^ Department of Molecular, Cellular, and Biomedical Sciences, University of New Hampshire, 46 College Road, Durham, NH 03824, USA

^8^ ISCARE IVF a.s. Jankovcova 1692, CZ-160 00 Praha 6

^9^ VFN Gynekologicko-porodnická klinika, Apolinářská 18, CZ-120 00, Czech Republic

*Corresponding authors: [falk@ibp.cz](mailto:falk@ibp.cz); [krat@fzu.cz](mailto:krat@fzu.cz)

**Chromatin architecture changes and DNA replication fork collapse are critical features in cryopreserved cells that are differentially controlled by cryoprotectants**

**RAW DATA**

[Type a quote from the document or the summary of an interesting point. You can position the text box anywhere in the document. Use the Drawing Tools tab to change the formatting of the pull quote text box.]


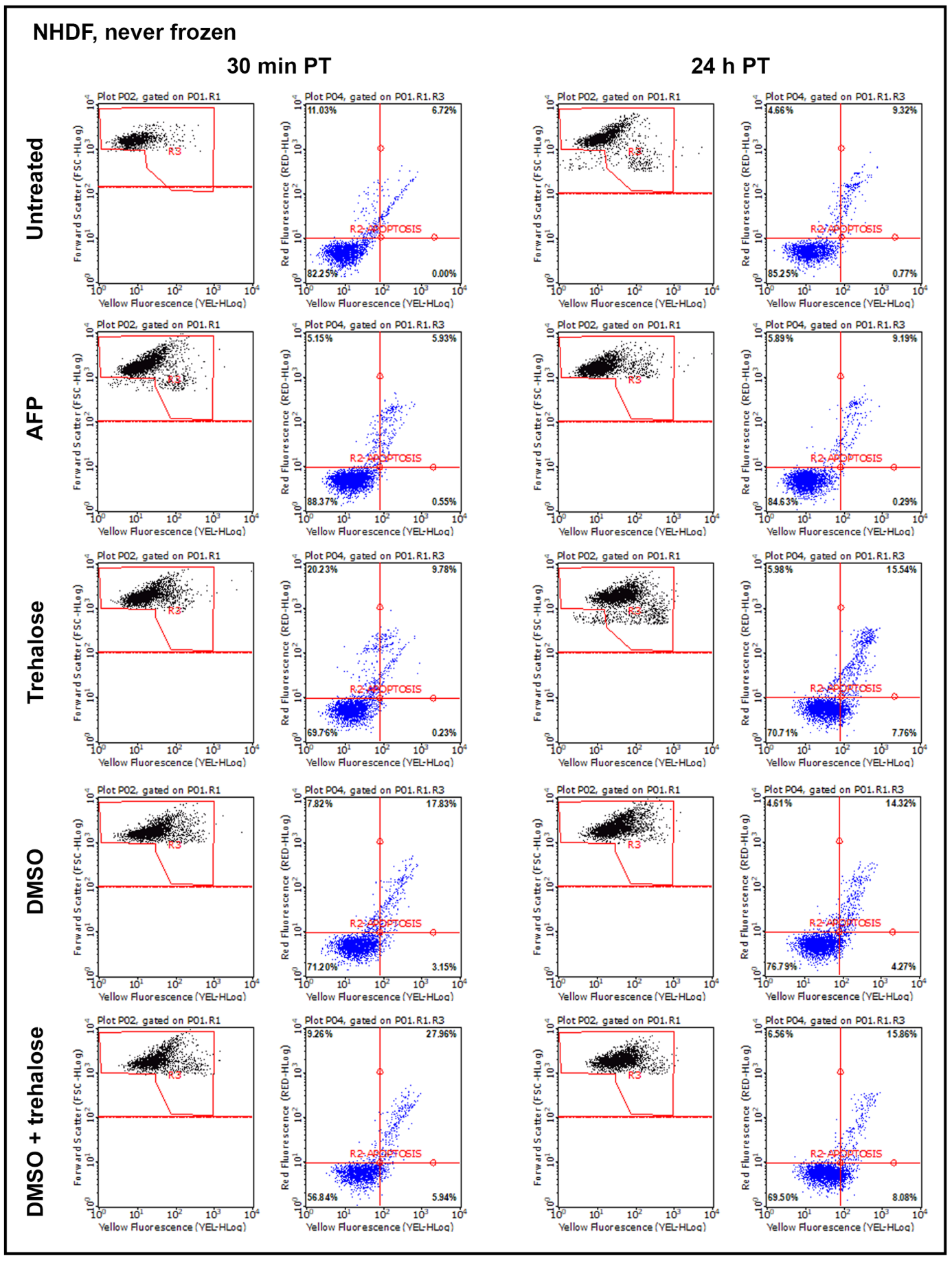
**Data 1A:** **Flowcytograms (Annexin V vs. Propidium iodide, PI) obtained for never frozen NHDF cells before (untreated) and 30 min or 24 h after their treatments (post-treatment, PT) with cryoprotectants (AFP, trehalose, DMSO or combination of trehalose + DMSO).** Left columns: FSC vs. Annexin V (YEL-HLog, horizontal axis) plots showing fractions of gated cells; right plots: Annexin V (YEL-HLog, horizontal axis) vs. Propidium iodide (PI, RED-HLog, vertical axis) showing cell viability. The percentage of cells is indicated for each Annexin V/PI category.


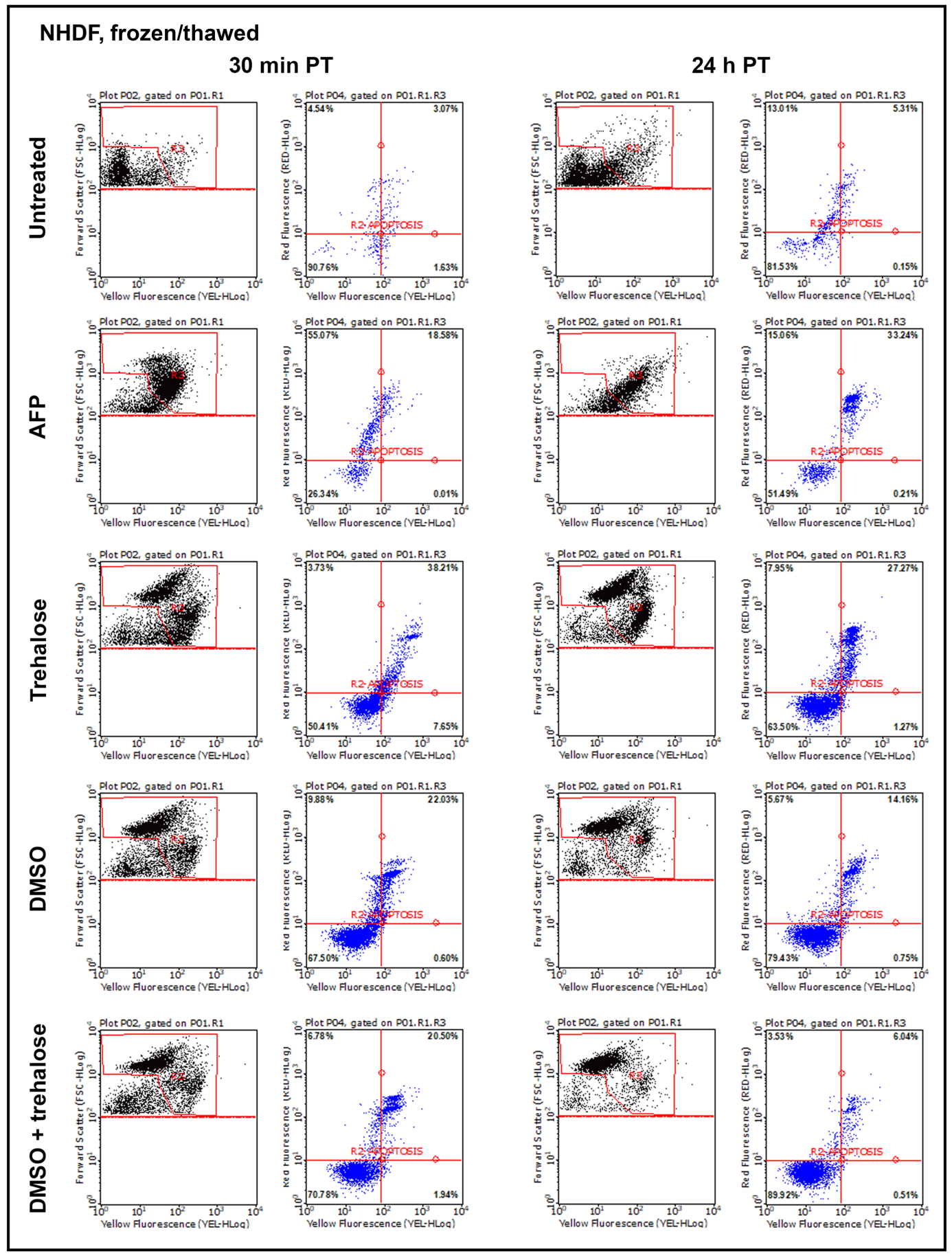
**Data 1B:** **Flowcytograms (Annexin V vs. Propidium iodide, PI) obtained for frozen/thawed NHDF cells immediately (30 min) and 24 h after their treatments with cryoprotectants (AFP, trehalose, DMSO or combination of trehalose + DMSO) and freezing/thawing.** Left columns: FSC vs. Annexin V (YEL-HLog, horizontal axis) plots showing fractions of gated cells; right plots: Annexin V (YEL-HLog, horizontal axis) vs. Propidium iodide (PI, RED-HLog, vertical axis) showing cell viability. The percentage of cells is indicated for each Annexin V/PI category.


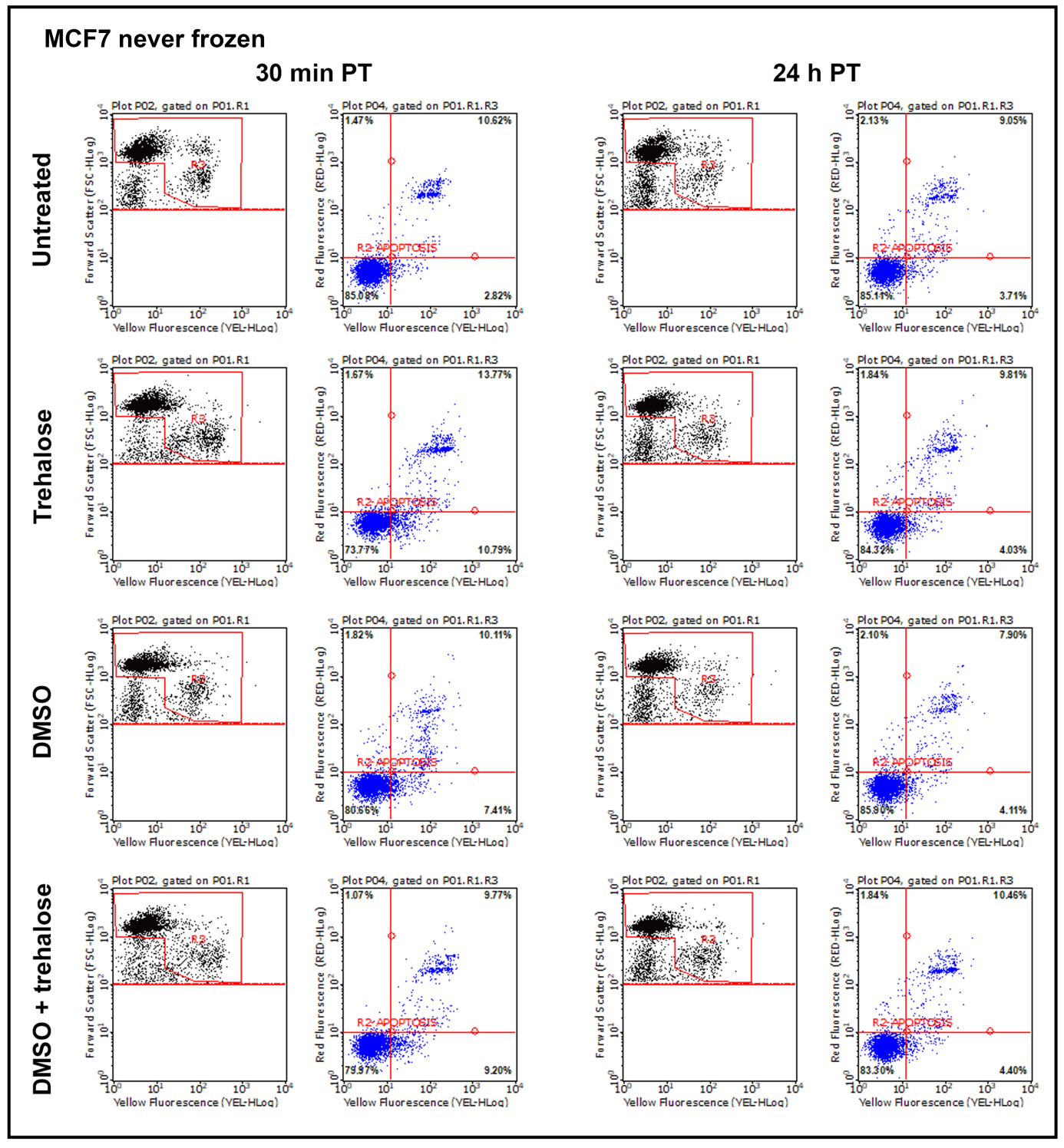


**Data 1C:** **Flowcytograms (Annexin V vs. Propidium iodide, PI) obtained for never frozen MCF7 cells before (untreated) and 30 min or 24 h after their treatments (post-treatment, PT) with cryoprotectants (AFP, trehalose, DMSO or combination of trehalose + DMSO).** Left columns: FSC vs. Annexin V (YEL-HLog, horizontal axis) plots showing fractions of gated cells; right plots: Annexin V (YEL-HLog, horizontal axis) vs. Propidium iodide (PI, RED-HLog, vertical axis) showing cell viability. The percentage of cells is indicated for each Annexin V/PI category.


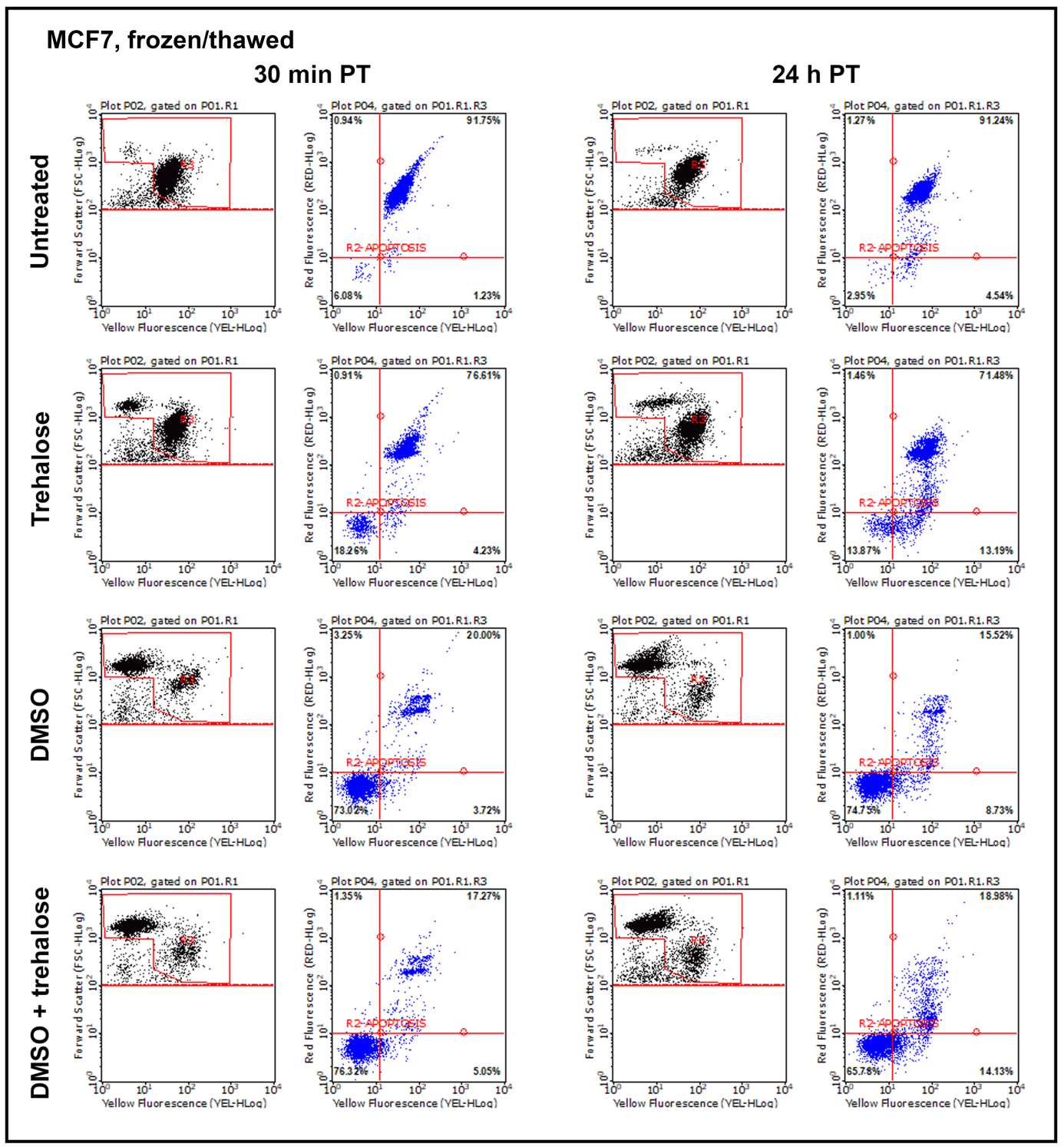


**Data 1D:** **Flowcytograms (Annexin V vs. Propidium iodide, PI) obtained for frozen/thawed MCF7 cells immediately (30 min) and 24 h after their treatments with cryoprotectants (AFP, trehalose, DMSO or combination of trehalose + DMSO) and freezing/thawing.** Left columns: FSC vs. Annexin V (YEL-HLog, horizontal axis) plots showing fractions of gated cells; right plots: Annexin V (YEL-HLog, horizontal axis) vs. Propidium iodide (PI, RED-HLog, vertical axis) showing cell viability. The percentage of cells is indicated for each Annexin V/PI category.


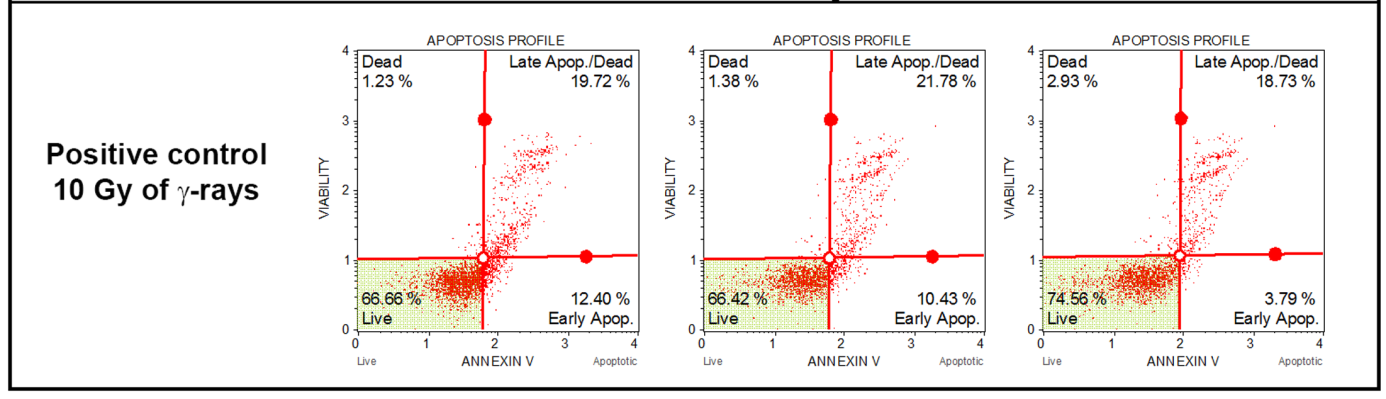


**Data 2:** **Flowcytograms (Annexin V vs. Propidium iodide, PI) obtained for positive controls – NHDF cells irradiated with 10 Gy of γ-rays, 24 h after their irradiation.** Annexin V (YEL-HLog, horizontal axis) vs. Propidium iodide (PI, RED-HLog, vertical axis) plots show cell viability. The percentage of cells is indicated for each Annexin V/PI category.


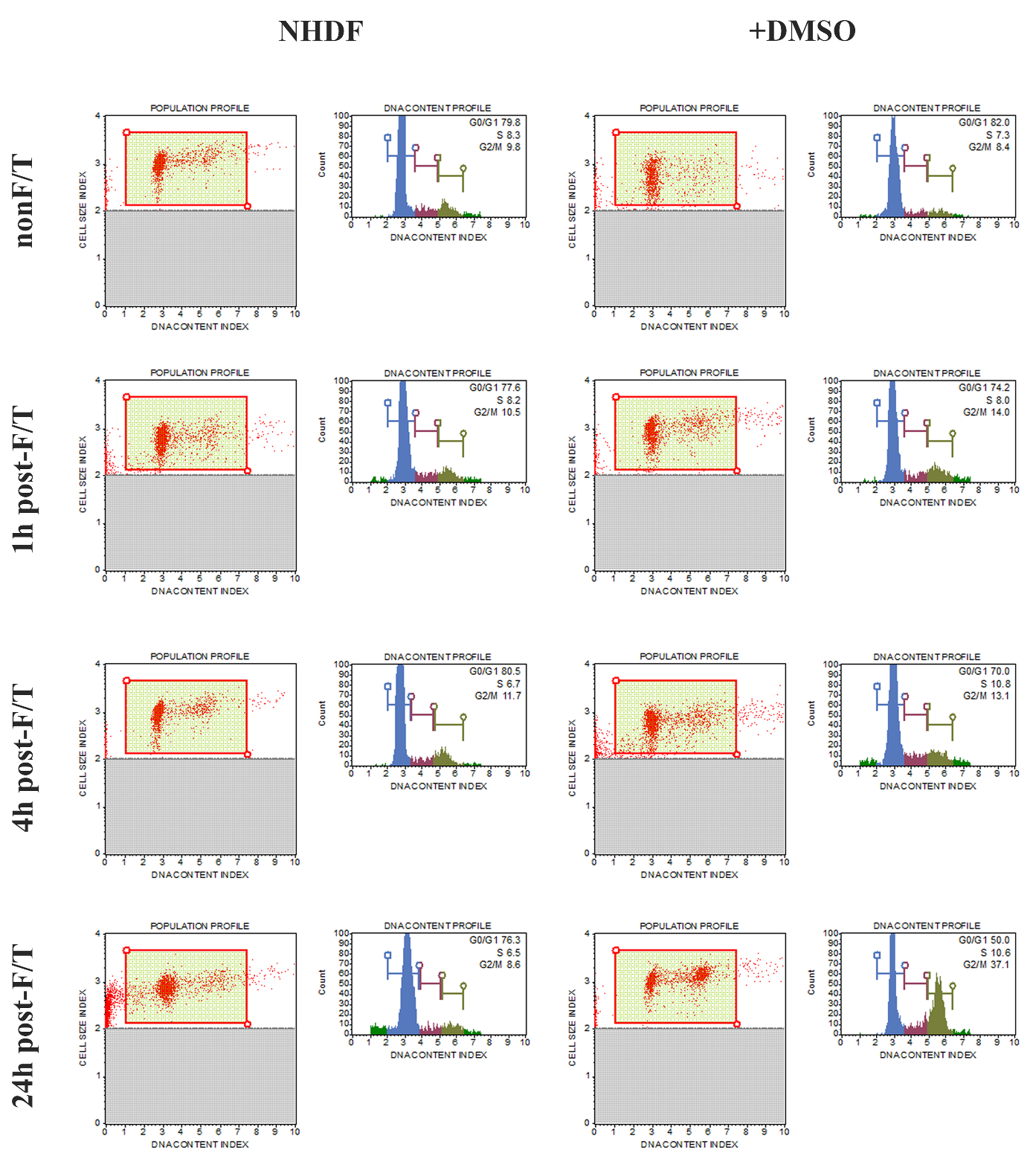


**Data 3 (part 1): Cell cycle distributions before and after freeing/thawing – flowcytograms for NHDF fibroblasts.** All cells harvested from cell cultures (including cells floating in the media) were scored according to DNA content before freezing (nonF/T) and 1, 4, and 24 hours after freezing/thawing (F/T).


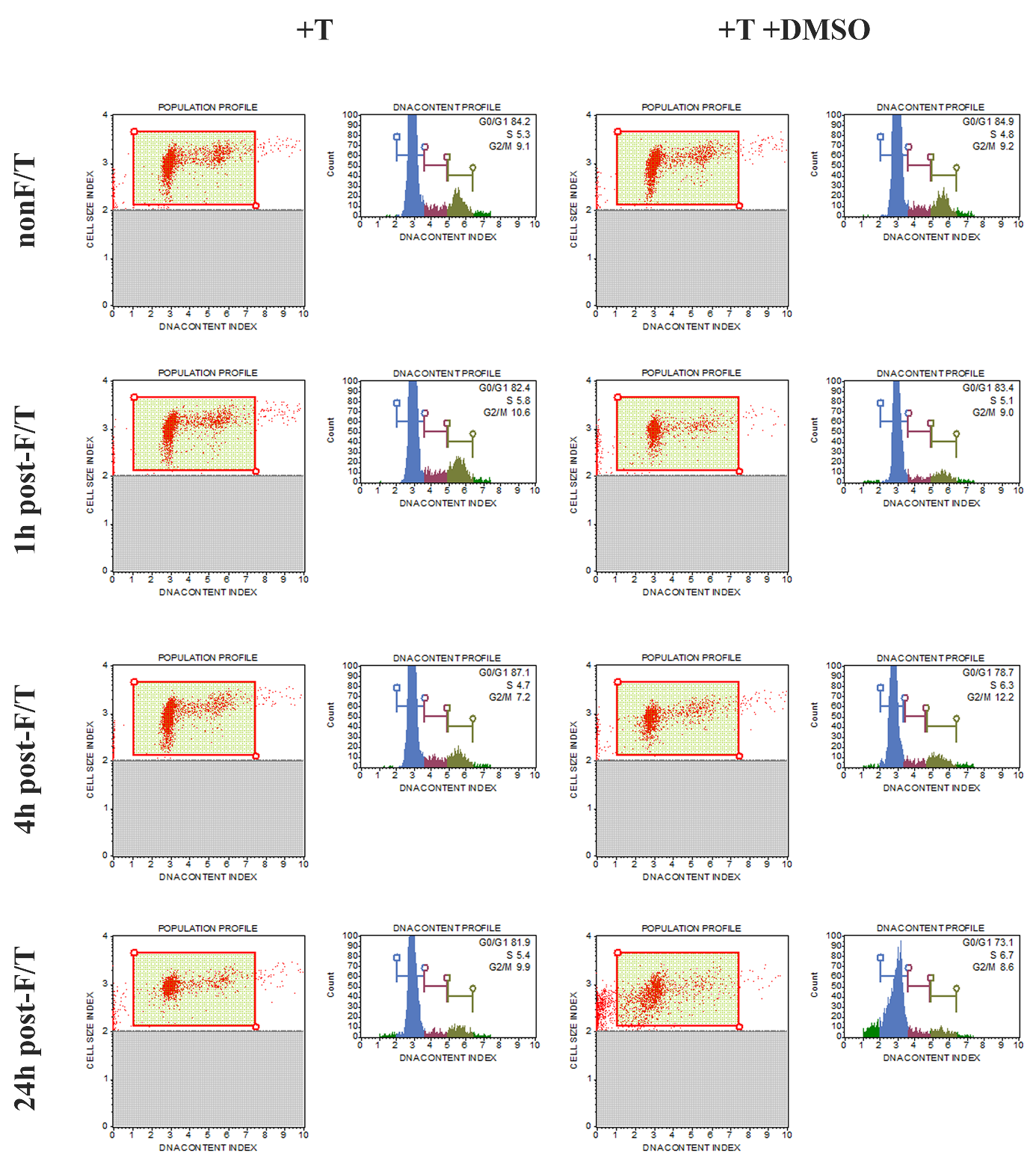


**Data 3 (part 2): Cell cycle distributions before and after freeing/thawing – flowcytograms for NHDF fibroblasts.** All cells harvested from cell cultures (including cells floating in the media) were scored according to DNA content before freezing (nonF/T) and 1, 4, and 24 hours after freezing/thawing (F/T).


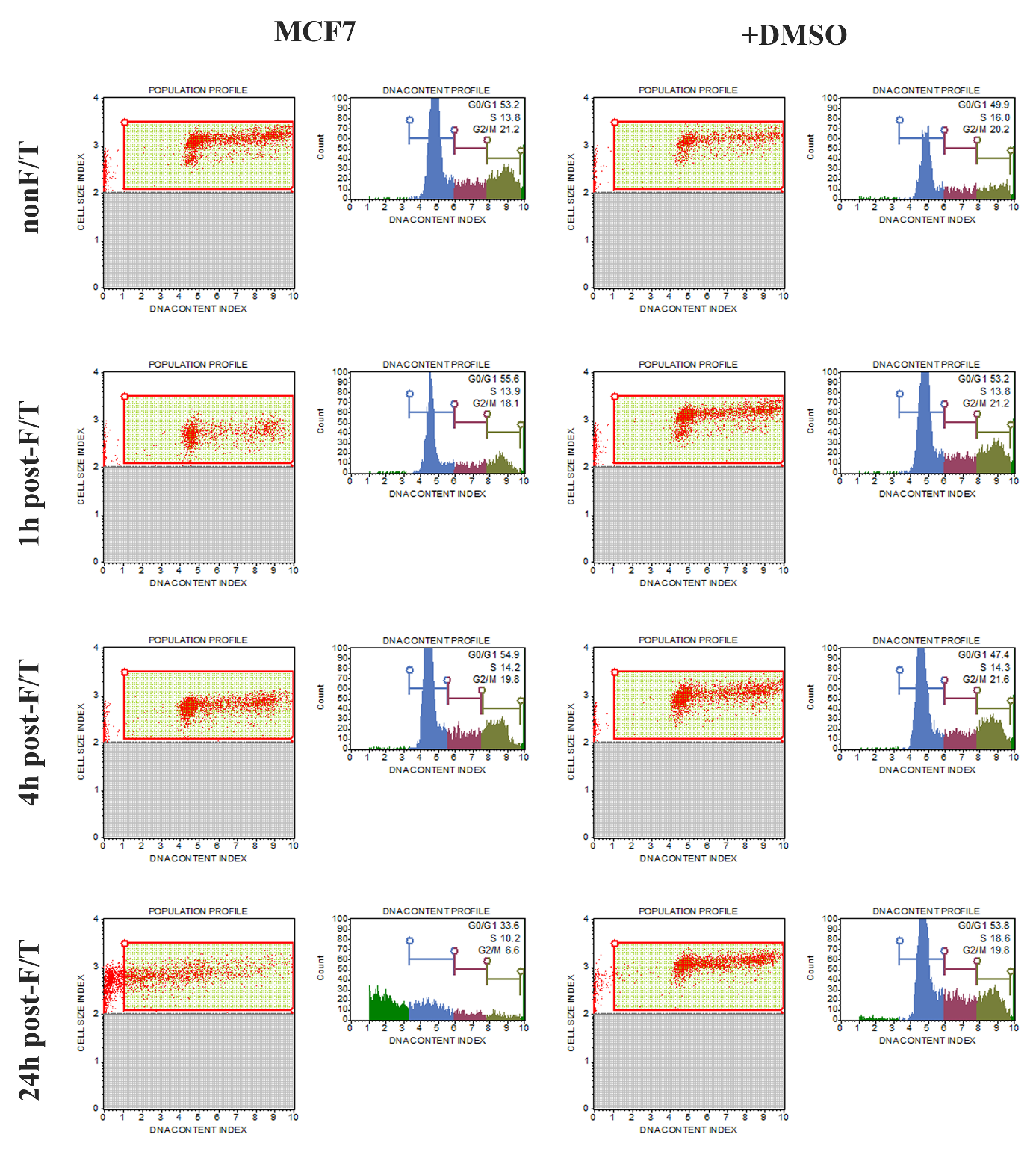


**Data 4 (part 1): Cell cycle distributions before and after freeing/thawing – flowcytograms for MCF7 cells.** All cells harvested from cell cultures (including cells floating in the media) were scored according to DNA content before freezing (nonF/T) and 1, 4, and 24 hours after freezing/thawing (F/T).


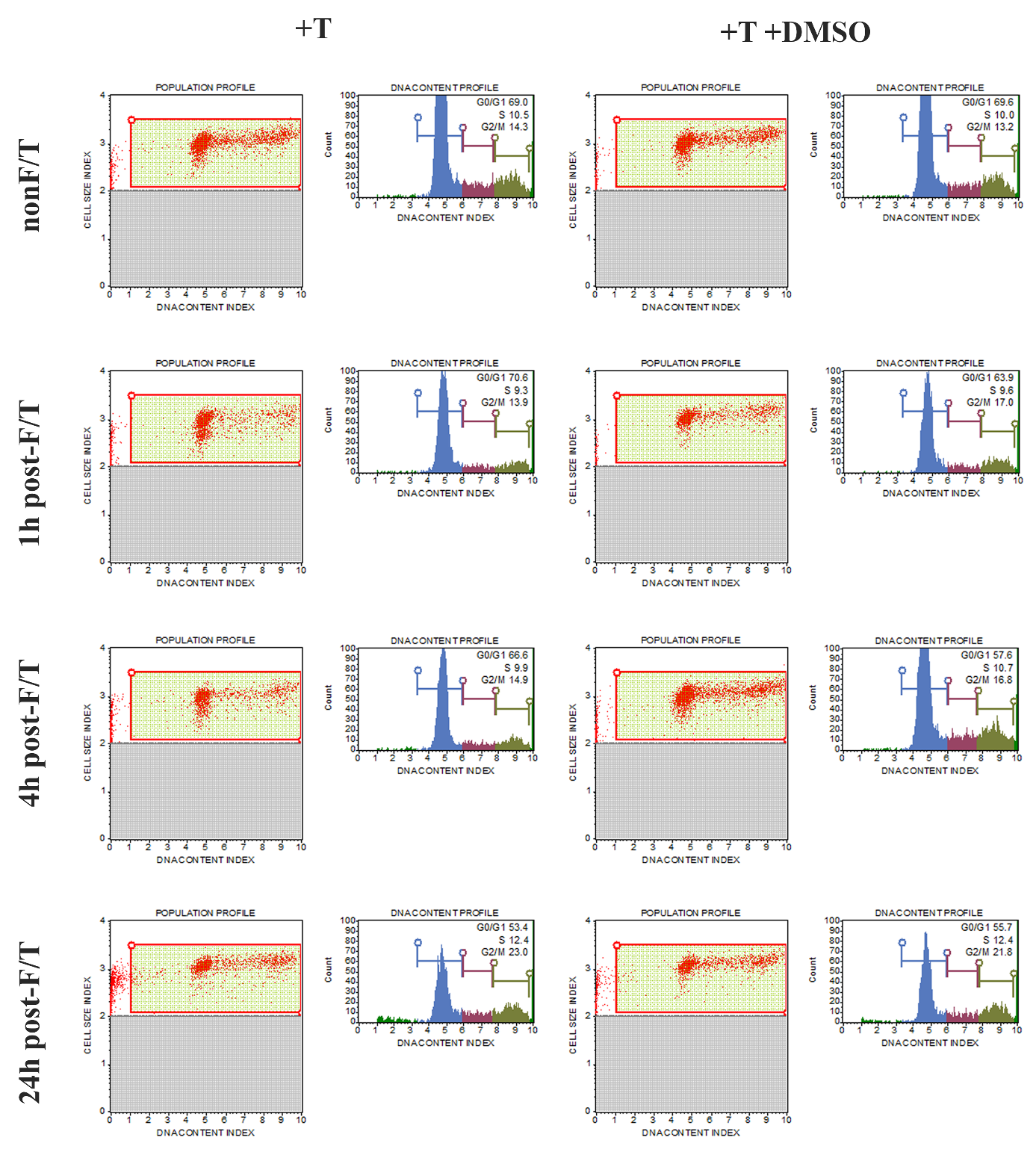


**Data 4 (part 2): Cell cycle distributions before and after freeing/thawing – flowcytograms for MCF7 cells.** All cells harvested from cell cultures (including cells floating in the media) were scored according to DNA content before freezing (nonF/T) and 1, 4, and 24 hours after freezing/thawing (F/T).


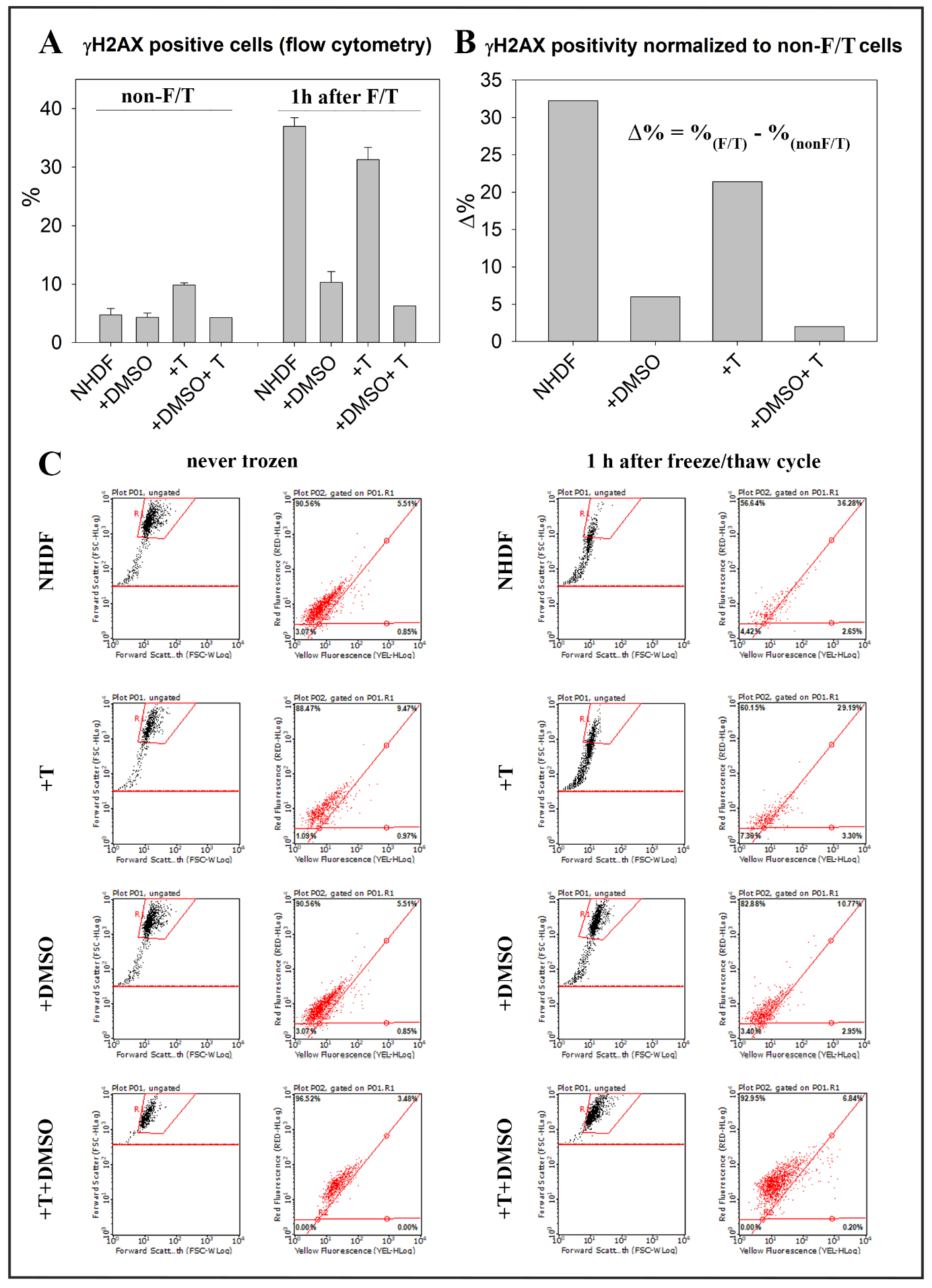
**Data 5: H2AX phosphorylation at Ser139 (γH2AX) quantified by flow cytometry in never frozen and frozen/thawed NHDF cells treated or not treated with cryoprotectants of different classes – trehalose, DMSO and their combination.** **A.** Proportions [%] of γH2AX-positive cells; **B.** Increase of γH2AX-positivity upon the freeze/thaw cycle, normalized to background values in non-frozen cells (calculated as Δ% = %_(F/T)_ - %_(nonF/T)_). **C.** Illustrative flowcytograms for all treatments; left: cell gating; right: flowcytograms showing γH2AX (YEL-HLog, horizontal axis) vs. H2AX (RED-HLog, vertical axis) cell staining intensity; the percentage of γH2AX positive cells is indicated (upper right corner).
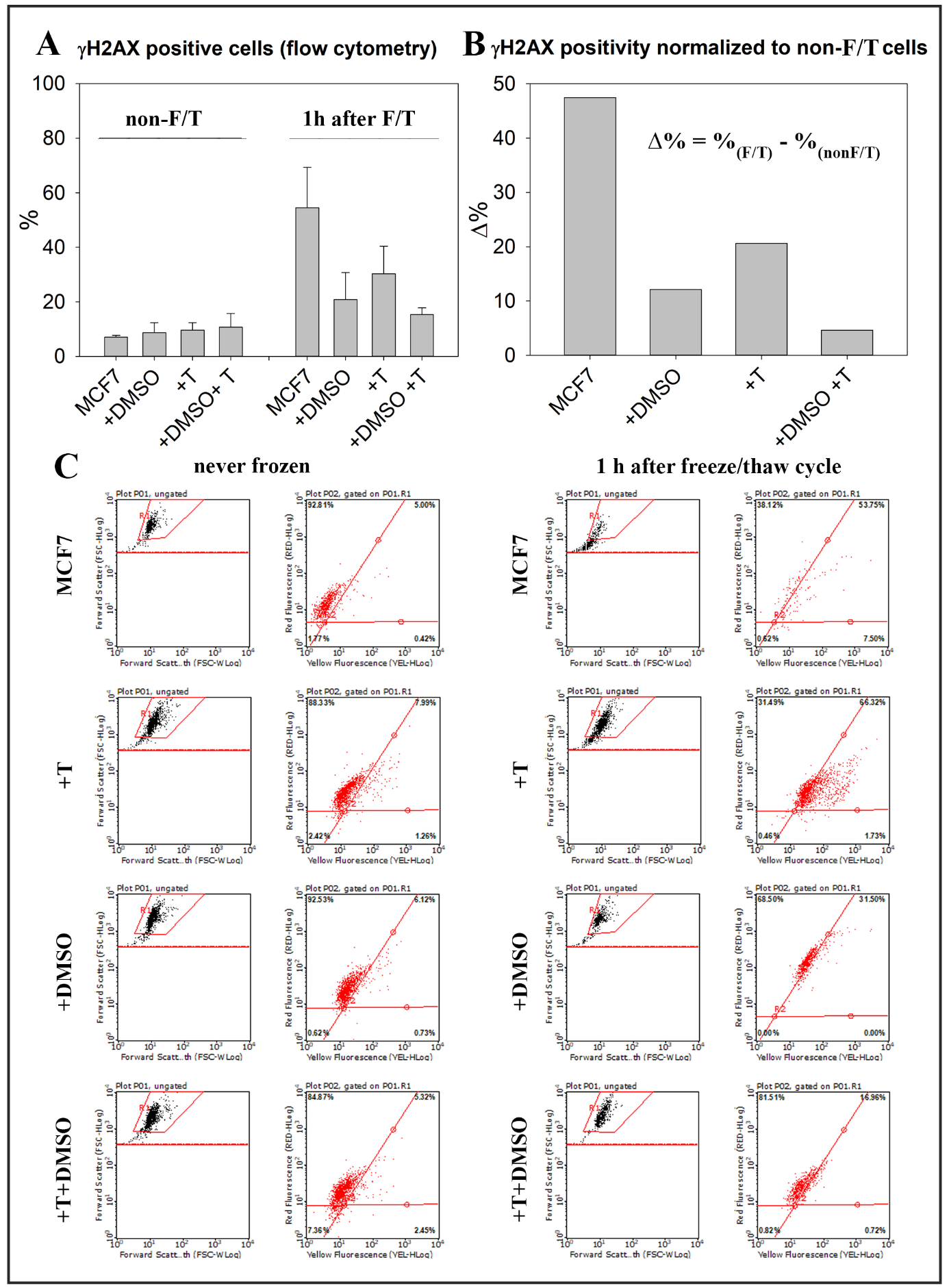
**Data 6: H2AX phosphorylation at Ser139 (γH2AX) quantified by flow cytometry in never frozen and frozen/thawed MCF7 cells treated or not treated with cryoprotectants of different classes – trehalose, DMSO and their combination.** **A.** Proportions [%] of γH2AX-positive cells; **B.** Increase of γH2AX-possitivity upon the freeze/thaw cycle, normalized to background values in non-frozen cells (calculated as Δ% = %_(F/T)_ - %_(nonF/T)_). **C.** Illustrative flowcytograms for all treatments; left: cell gating; right: flowcytograms showing γH2AX (YEL-HLog, horizontal axis) vs. H2AX (RED-HLog, vertical axis) cell staining intensity; the percentage of γH2AX positive cells is indicated (upper right corner).
